# Exploring the relationship between metabolism and immune microenvironment in osteosarcoma based on metabolic pathways

**DOI:** 10.1186/s12929-024-00999-7

**Published:** 2024-01-12

**Authors:** Changwu Wu, Jun Tan, Hong Shen, Chao Deng, Christian Kleber, Georg Osterhoff, Nikolas Schopow

**Affiliations:** 1 Department of Neurosurgery, Xiangya Hospital, Central South University, Changsha, Hunan China; 2 National Clinical Research Center for Geriatric Disorders, Xiangya Hospital, Central South University, Changsha, Hunan China; 3 Department of Oncology, Xiangya Hospital, Central South University, Changsha, Hunan China; 4 Key Laboratory for Molecular Radiation Oncology of Hunan Province, Xiangya Hospital, Central South University, Changsha, Hunan China; 5 Department of Orthopedics, Xiangya Hospital, Central South University, Changsha, Hunan China; 6 Sarcoma Center, Department of Orthopedics, Trauma and Plastic Surgery, University Hospital Leipzig, Leipzig, Germany

**Keywords:** Osteosarcoma, Metabolism, Tumor immune microenvironment, Prognosis, Vitamin and cofactor, Treatment response, ST3GAL4

## Abstract

**Background:**

Metabolic remodeling and changes in tumor immune microenvironment (TIME) in osteosarcoma are important factors affecting prognosis and treatment. However, the relationship between metabolism and TIME needs to be further explored.

**Methods:**

RNA-Seq data and clinical information of 84 patients with osteosarcoma from the TARGET database and an independent cohort from the GEO database were included in this study. The activity of seven metabolic super-pathways and immune infiltration levels were inferred in osteosarcoma patients. Metabolism-related genes (MRGs) were identified and different metabolic clusters and MRG-related gene clusters were identified using unsupervised clustering. Then the TIME differences between the different clusters were compared. In addition, an MRGs-based risk model was constructed and the role of a key risk gene, ST3GAL4, in osteosarcoma cells was explored using molecular biological experiments.

**Results:**

This study revealed four key metabolic pathways in osteosarcoma, with vitamin and cofactor metabolism being the most relevant to prognosis and to TIME. Two metabolic pathway-related clusters (C1 and C2) were identified, with some differences in immune activating cell infiltration between the two clusters, and C2 was more likely to respond to two chemotherapeutic agents than C1. Three MRG-related gene clusters (GC1-3) were also identified, with significant differences in prognosis among the three clusters. GC2 and GC3 had higher immune cell infiltration than GC1. GC3 is most likely to respond to immune checkpoint blockade and to three commonly used clinical drugs. A metabolism-related risk model was developed and validated. The risk model has strong prognostic predictive power and the low-risk group has a higher level of immune infiltration than the high-risk group. Knockdown of ST3GAL4 significantly inhibited proliferation, migration, invasion and glycolysis of osteosarcoma cells and inhibited the M2 polarization of macrophages.

**Conclusion:**

The metabolism of vitamins and cofactors is an important prognostic regulator of TIME in osteosarcoma, MRG-related gene clusters can well reflect changes in osteosarcoma TIME and predict chemotherapy and immunotherapy response. The metabolism-related risk model may serve as a useful prognostic predictor. ST3GAL4 plays a critical role in the progression, glycolysis, and TIME of osteosarcoma cells.

**Supplementary Information:**

The online version contains supplementary material available at 10.1186/s12929-024-00999-7.

## Introduction

Osteosarcoma is a malignant bone tumor that predominantly affects children and young adults. Despite advances in treatment, the prognosis for patients with osteosarcoma remains poor, with a 5-year survival rate of approximately 60–70% for localized disease and less than 30% for metastatic disease [1]. The development and progression of osteosarcoma is a complex process that involves multiple molecular and cellular mechanisms. In recent years, there has been increasing interest in the role of metabolic reprogramming and the tumor immune microenvironment (TIME) in osteosarcoma, and their potential as therapeutic targets.

Tumor cells have to modify their metabolic program to support the energy and macronutrient requirements of rapid proliferation. Metabolic reprogramming is now recognized as a hallmark of cancer and is one of the most critical biological differences between tumor cells and normally differentiated cells [2]. For example, in many tumor cells, altered carbohydrate metabolism, represented by the Warburg effect, provides a proliferative advantage for tumor cells [3]. Osteosarcoma cells exhibit a variety of metabolic alterations, including increased glucose uptake, altered mitochondrial function, and increased reliance on glycolysis for ATP generation [4]. These metabolic changes are driven by a variety of signaling pathways, including the PI3K/AKT/mTOR pathway and the HIF-1α pathway [5]. They provide potential therapeutic targets for the treatment of osteosarcoma, as inhibition of key metabolic pathways could potentially starve cancer cells of the nutrients they need to proliferate.

TIME plays a critical role in a variety of biological processes including proliferation, metastasis, and treatment response (including chemotherapy, radiation therapy and immunotherapy) in osteosarcoma [6–8]. Previous studies have shown that patients with different TIME status within their osteosarcoma have very different prognoses [9]. Specifically, patients with “hot” tumors that have more immune cell infiltration in TIME have a better prognosis, while patients with “cold” tumors that have less immune cell infiltration have a worse prognosis. Therefore, therapeutic agents that modulate TIME, transform “cold” tumors into “hot” tumors, and use existing immunity to destroy osteosarcoma cells are increasingly being considered as new options with great potential for application [10, 11]. Indeed, recent studies have proven that immunotherapy has shown advantages over conventional interventional strategies in inhibiting osteosarcoma metastasis and recurrence, and satisfactory efficacy in inhibiting the progression of advanced osteosarcoma [12–14].

The interaction between tumor metabolism and immunity has been intensively studied and it is generally recognized that oncogenic transformation can lead to the adaptation of a well-characterized metabolic phenotype in cancer cells that can profoundly affect the TIME [15]. Specifically, in addition to affecting cancer cells directly, metabolic reprogramming of tumors also alters the TIME by affecting the behavior of other cell types, such as immune cells and stromal cells [16]. For example, the acidic microenvironment created by aerobic glycolysis can suppress the immune system and promote the growth of blood vessels, which can in turn promote tumor growth and metastasis [17]. In particular, the lactate produced by aerobic glycolysis can induce the infiltration of regulatory T cells and the M2 polarization of macrophages in tumors, thereby promoting immune suppression [18]. Tumor metabolic heterogeneity refers to the significant differences in metabolic characteristics that exist between different tumors or within the same tumor tissue. It is an important aspect of tumor heterogeneity and is mainly driven by different genotypes or microenvironments [19–21]. The research by Feng et al. demonstrates that within the same type of tumor, patients can be divided into subgroups suitable for different treatment methods based on different metabolic characteristics [22]. Therefore, identifying the metabolic profile of different osteosarcoma patients will not only explore the impact of different metabolic landscapes on TIME, but also guide treatment decisions. However, few studies have been conducted to genomically analyze osteosarcoma from a global perspective of metabolic heterogeneity. The few previous studies have been limited to a specific metabolic pathway [23, 24]. In this study, we focused on the seven most prominent metabolic super-pathways with the aim of comprehensively assessing metabolic pathways of prognostic importance in osteosarcoma, identifying tumor subtypes with different metabolic profiles and exploring the heterogeneity of TIME profiles and treatment response across tumor subtypes.

## Methods and materials

### Data acquisition, clinical samples and cell lines

Standardized RNA-Seq data and clinical information for 88 independent osteosarcoma samples from the TARGET database were obtained from Xena Functional Genomics Explorer (http://xena.ucsc.edu/), of which 84 samples with complete survival information were included in this study. In addition, standardized microarray expression data and clinical information for 34 osteosarcoma samples from the GSE16091 cohort were obtained from the GEO database (https://www.ncbi.nlm.nih.gov/geo/) [25]. The gene expression data distribution from the TARGET and GEO databases was analyzed using the PCA algorithm, and it was found that there were no apparent batch effects in the data (Additional file 1: Figure S1). In addition, gene sets for seven metabolic super-pathways annotated according to the Reactome database were collected from a previous study (Additional file 2: Table S1) [26]. Together, these gene sets represent the major metabolic processes, including amino acid metabolism, carbohydrate metabolism, integration of energy metabolism, lipid metabolism, nucleotide metabolism, tricarboxylic acid (TCA) cycle, and vitamin & cofactor metabolism.

Tumor samples of 14 primary osteosarcoma patients who underwent surgical resection between 2018 and 2019 and 5 normal tissue samples were obtained from Xiangya Hospital, Central South University, Hunan, China. All samples were evaluated by pathologists and preserved in paraffin. Only relapse-free survival (RFS) data is currently available, as most patients are still alive. The collection of human tissues was approved by the Medical Ethics Committee of Xiangya Hospital of Central South University (Approval number: 202303046).

U2OS, MG-63 and THP-1 cell lines were obtained from the Xiangya cell repository and U2OS and MG-63 were cultured in Dulbecco’s modified Eagle’s medium (DMEM, Biological Industries, Israel) containing 10% fetal bovine serum (Gibco, USA) at 5% CO2 and 37 °C. THP-1 cell line was cultured in RPMI 1640 medium (Gibco, Thermo Fisher Scientific, USA). THP-1 cells were differentiated into M0 macrophages by incubation with 100 ng/mL phorbol 12-myristate 13-acetate for 24 h. Typical images of THP-1 cells, M0 macrophages, and M2 macrophages were shown in Additional file 1: Figure S2. The ST3GAL4 overexpression plasmid was synthesized by Sino Biological (Beijing, China) and the overexpression efficiency was shown in Additional file 1: Figure S3. The small interfering RNA si-ST3GAL4 and the empty vector si-NC were synthesized by GenePharma (Shanghai, China). A total of three siRNAs were validated, among which siRNA-2 showed the highest efficiency and was used for subsequent experiments (Additional file 1: Figure S4).

### Pathway enrichment analysis

To quantify the activity of the seven metabolic pathways in a single tumor sample, gene set variation analysis (GSVA) was performed using the R package “GSVA” to calculate the enrichment score of each pathway in the individual sample. Subsequently, Kaplan- Meier curve and log-rank test were used to explore the relationship between the seven metabolic pathways and overall survival (OS) of patients with osteosarcoma. The “surv_cutpoint” function of the “survminer” R package was used to determine the optimal cut-off point of each metabolic pathway based on the maximally selected log-rank statistics. In addition, a set of core biological pathway gene sets closely related to tumors was collected from the study of Mariathasan et al. and the activity of each pathway was calculated by GSVA [27]. This includes three epithelial mesenchymal transition (EMT) signatures originating from different publications and composed of different genes. The correlation of key metabolic pathways with core biological pathways was then calculated using Spearman’s correlation analysis. Gene set enrichment analysis (GSEA) between the two groups of samples was performed in Sangerbox (http://vip.sangerbox.com/) using the GSEA software (Version 4.1.0) based on the HALLMARK and KEGG gene sets [28].

### Identification of hub genes in the vitamin & cofactor metabolic pathway

The protein–protein interaction (PPI) network was constructed using the STRING database (https://string-db.org/) and visualized using the Cytoscape software (Version 3.8.2). The hub genes of the vitamin & cofactor metabolic pathway were then identified based on the PPI network using the “cytohubba” plugin in Cytoscape [29]. Gene modules in the vitamin & cofactor metabolic pathway were analyzed using the “MCODE” plugin. After matching the hub genes with RNA-Seq data, univariate Cox regression analysis was performed to determine the effect of the hub genes on OS in patients with osteosarcoma. In addition, similar methods were used to analyze the hub genes of other metabolic super-pathways.

### Identification of metabolic pathway-related clusters

After identifying key metabolic pathways in osteosarcoma, PAM-based unsupervised consensus clustering was used to identify potential metabolic subtypes as we described previously [30–32]. In brief, 1000 bootstraps were performed and K value was set to 2–10, and the optimal number of clusters was defined by the consensus cumulative distribution function and the consensus heatmap. This method was also used for the identification of clusters based on seven metabolic pathways.

### Immune infiltration analysis

The ESTIMATE algorithm is a method for inferring the overall level of immune infiltration in tumor tissues based on gene expression data and has been widely used in a large number of previous studies [33, 34]. This study used this algorithm to infer the ImmuneScore, StromalScore and ESTIMATEScore (inversely correlated with tumor purity) of patients with osteosarcoma. In addition, the single sample GSEA (ssGSEA) method was used to infer the levels of 28 immune cell infiltration in osteosarcoma based on a previous report [35].

### Identification of metabolism-related genes (MRGs) in osteosarcoma

Using the R package “limma” to compare differentially expressed genes between different metabolic pathway-related clusters, the threshold was set to *p* < 0.05 and log_2_|fold change|> 0.25. These genes were considered as MRGs. Subsequently, Gene Ontology (GO) enrichment analysis and Kyoto Encyclopedia of Genes and Genomes (KEGG) pathway analysis were performed on MRGs. The prognostic role of MRGs in patients with osteosarcoma was analyzed using univariate Cox regression. After obtaining prognosis-related MRGs, potential MRG-associated gene clusters were identified using unsupervised clustering analysis.

### Construction of the risk model

In this study, prognosis-related MRGs were downscaled and hub prognostic genes were obtained using the Least Absolute Shrinkage and Selection Operator (LASSO) regression analysis. Subsequently, the hub prognostic genes were placed into the stepwise multivariate regression to construct risk models, and the risk model with the largest C-index was considered the best risk model. The risk model was calculated using the following formula:

$$risk\, score=\sum {k}_{j}\times {Exp}_{i}$$

where k_j_ is the coefficient of each gene in the risk model, and Exp_i_ is the gene expression. The prediction accuracy of the risk score was quantified by drawing ROC curves using the “timeROC” R package. This R package is widely used to estimate time-dependent ROC curves and the area under the time-dependent ROC curve (AUC) in the presence of censoring data [36]. The package uses the inverse probability of censoring weighting method to estimate and handle censoring data.

### Analysis of drug sensitivity and response to immunotherapy

As previously described [37], normalized gene expression data of 809 tumor cell lines and response data for each cell line to three guideline-based used chemotherapeutic agents (cisplatin, cyclophosphamide and gemcitabine) for osteosarcoma and one targeted agent (sorafenib) with clinical application value were downloaded from the Drug Sensitivity in Cancer (GDSC) database, and the drug response data were converted to the IC_50_. Then the IC_50_ of every drug in individual osteosarcoma patient was estimated based on oncoPredict algorithm using the gene expression profile of these cell lines and drug response data as the training set. Maeser et al. provided a detailed explanation of the usage details of the oncoPredict algorithm [38]. Jiang et al. developed TIDE using RNA-Seq data from tumors treated with anti-PD1 and anti-CTLA4 therapies, and identified it as an effective predictor of the responsiveness to these therapies [39]. In this study, it was used to infer the response of osteosarcoma patients to immune checkpoint blockade (ICB). The TIDE score is based on two mechanisms of tumor immune escape, including the dysfunction of tumor-infiltrating cytotoxic T lymphocytes (CTL) and the exclusion of CTLs by immunosuppressive factors, and three cell types that limit T cell infiltration in tumors, including the tumor-associated fibroblasts (CAF), myeloid-derived suppressor cells (MDSC), and M2 tumor-associated macrophages (TAM_M2).

### Single-cell RNA-sequencing (scRNA-seq) analysis

The scRNA-seq dataset GSE152048 containing 11 osteosarcoma samples was downloaded from the GEO database [40]. The dataset was processed and analyzed according to the standard procedure of the R package “Seurat” (v.4.3.0), and a total of 26,175 genes and 123,322 cells were included in the study. After performing data downscaling and clustering, the clusters were annotated using the previously reported cellular markers [40]. In addition, the expression of ST3 beta-galactoside alpha-2,3-sialyltransferase 4 (ST3GAL4) in the scRNA-seq dataset GSE162454 was also analyzed using the Tumor Immune Single-cell Hub (TISCH) (http://tisch.comp-genomics.org/) [41].

### Quantitative reverse transcription‐PCR (RT‐qPCR)

Adding 1 ml TriPure, chloroform, isopropanol and 75% anhydrous ethanol to the 6-well plate to extract cell RNA. After quantification, RNA reverse transcription and RT-qPCR were performed as described previously [42]. The PCR primers used are listed in Additional file 3: Table S2.

### Cell proliferation assay

After cell transfection using the Lipofectamine^®^ 3000 (Invitrogen, Carlsbad, CA, USA) according to the manufacturer’s instructions, the transfected cells were cultured for 24 h and inoculated in 96-well plates with 2000 cells per well. After cell walling, cells were incubated for different time periods (0, 1, 2 and 3 days) and 10 µl of CCK-8 reagent was added to each well. After incubation at 37 °C for 3 h, absorbance was measured at 450 nm to determine cell viability.

### Cell invasion and migration assays

Cell invasion ability was measured using the Transwell assay and cell migration ability was measured using the scratch assay. The Transwell and scratch assays were carried out as described previously [9]. The scratch assay was incubated for 36 h. For Cell invasion assay, the cells were resuspended in serum-free medium and placed in the upper chamber of the Transwell system. Culture medium with 10% serum was added to the lower chamber and was used as a chemoattractant. After 24 h of incubation, the cells were stained and counted.

### Colony formation assay

MG-63 and U2OS cells with knocked down or overexpressed ST3GAL4 were seeded in 6-well plates at a density of 1000 cells per well. After 10 days of cultivation, the cells were fixed with 4% paraformaldehyde at room temperature for 30 min. Subsequently, the cells were stained with 0.1% crystal violet, and the number of colonies in each well was counted.

### Seahorse assays

10,000 tumor cells were seeded in a Seahorse 96-well assay plate and incubated overnight. The probe plates were pretreated and the calibration solution was prepared following the manufacturer’s protocol. Subsequently, the probe plates were placed in a CO_2_-free incubator overnight. After the overnight incubation, the detection solution was prepared as per the instructions of the Glycolysis Stress Test kit (Agilent Technologies, #103020-100) and the reagents were added sequentially. Real-time metabolic changes in cells were detected using the Agilent Seahorse XFe96 (Agilent Technologies).

### Co-culture experiment and flow cytometry assay

Co-culture was performed using the Boyden chamber, M0 macrophages were seeded at upper chamber and tumor cells were seeded at lower chamber. After 48 h, cells from the upper chamber were collected. For flow cytometry assay, cells were prepared for single cell suspension and were fixed with 2% paraformaldehyde solution in PBS.

Then, cells were fixed and permeabilized with the FIX & PERM Kit (MultiSciences Biotech, Hangzhou, China) and stained with CD206 (321104; Biolegend). A FACS flow cytometer (BD FACS LSRFortessa, USA) was used for the flow cytometry analysis.

### Immunohistochemistry (IHC)

IHC was carried out as described previously [43]. The rabbit polyclonal antibody to ST3GAL4 was purchased from Invitrogen (PA5-62056, 1:200 dilution). Two blinded pathologists scored the intensity and percentage of positive cells for ST3GAL4 staining. The intensity was scored as follows: 0 (negative), 1 (weakly positive), 2 (moderately positive), and 3 (strongly positive). The percentage of ST3GAL4-positive cells was scored as follows: 0 (0%), 1 (1–25%), 2 (26–50%) and 3 (> 50%). The IHC score was defined as the sum of the intensity score and the percentage score of positive cells.

### Statistical analysis

Differences between two groups were compared using unpaired Student's t-test or Wilcoxon rank sum test. For comparisons between more than two groups, differences were compared using one-way ANOVA or Kruskal–Wallis test. The correlation between two groups was calculated using Sperman’s correlation analysis. Unless otherwise indicated, statistical significance was set at two-sided *p* < 0.05. All statistical calculations were performed using R software (Version 4.2.1).

## Results

### Identification of key metabolic pathways in osteosarcoma

**Fig. 1:**
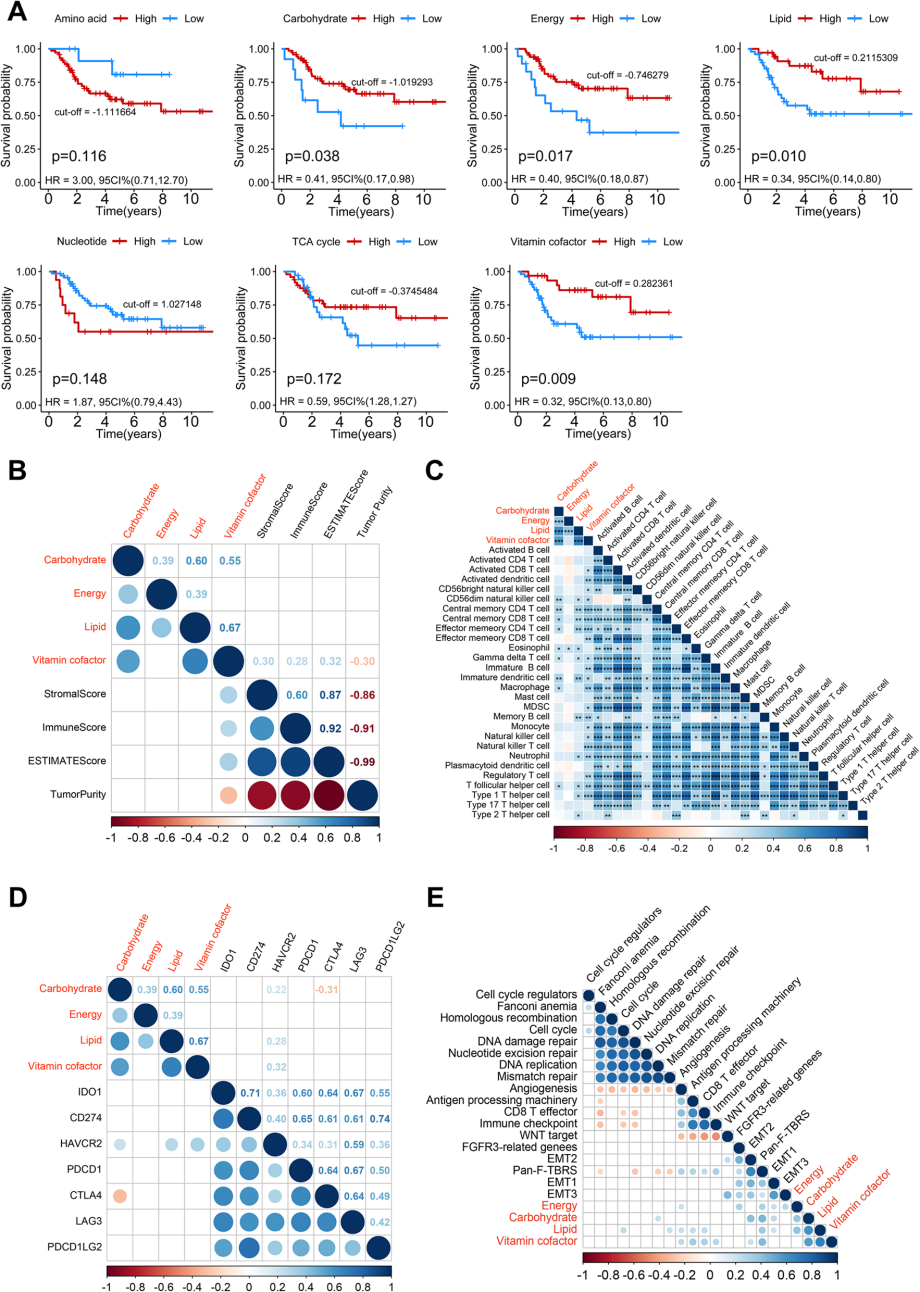
Identification of key metabolic pathways in osteosarcoma. **A** Kaplan–Meier curves depict the overall survival difference between pathway activity-high and pathway activity-low groups in the TARGET cohort. Red representing the pathway activity-high group and blue representing the pathway activity-low group. **B** Correlations between four key metabolic pathways and immune infiltration scores, only correlations that are significant are shown. **C** Correlations between four key metabolic pathways and abundance of 28 immune cells, only correlations that are significant are shown. **D** Correlations between four key metabolic pathways and expression of immune checkpoint genes. **E** Correlations between four key metabolic pathways and known core biological pathway scores. Correlation coefficients are calculated by Spearman’s correlation analysis, with red representing negative correlations and blue representing positive correlations. Blank represents a correlation P-value > 0.05

Metabolic heterogeneity may lead to differences in clinical outcomes, and we are committed to exploring the key metabolic pathways associated with clinical outcomes. After quantifying the activity of the seven metabolic super-pathways, Kaplan–Meier curve analysis identified four key metabolic pathways that were significantly associated with prognosis. Higher levels of carbohydrate (*p* = 0.038), energy (*p* = 0.017), lipid (*p* = 0.010), and vitamin & cofactor metabolism (*p* = 0.009) were associated with better OS in osteosarcoma (Fig. 1A). Among them, vitamin & cofactor metabolism has the highest significance. These four key metabolic pathways were first explored in relation to the overall level of immune infiltration. As shown in Fig. 1B, only vitamin & cofactor metabolism is significantly positively correlated with overall immune and stromal infiltration and negatively correlated with tumor purity. Further analysis of immune cell infiltration revealed a potential positive correlation between vitamin & cofactor metabolism and infiltration of most immune cells, including activated CD8 T cells and activated dendritic cell (Fig. 1C). Consistently, in the subgroup analysis, only the high vitamin & cofactor metabolism group showed higher levels of immune cell infiltration compared to the low vitamin & cofactor metabolism group (Additional file 1: Figure S5). Due to the positive correlation with vitamin & cofactor metabolism, carbohydrate and lipid metabolism were also positively correlated with infiltration of some immune cells. Immune checkpoint analysis only found a potential positive correlation between hepatitis A virus cellular receptor 2 (HAVCR2) and metabolic pathways (Fig. 1D). Moreover, a robust positive correlation prevails among diverse immune characteristics, encompassing immune checkpoints and the infiltration of immune cells (Additional file 1: Figure S6). Core biological pathway analysis revealed that vitamin & cofactor metabolism was potentially associated with immune-related biological pathways, such as CD8 T effector, antigen processing and immune checkpoint (Fig. 1E). The results of the subgroup analysis further confirmed these findings (Additional file 1: Figure S7). Lipid metabolism was associated with some immune pathways, but also with cell cycle and mismatch repair. Overall, vitamin & cofactor metabolism is the important metabolic pathway affecting prognosis and TIME in osteosarcoma.

### Identification of hub genes in the vitamin & cofactor metabolic pathway

**Fig. 2:**
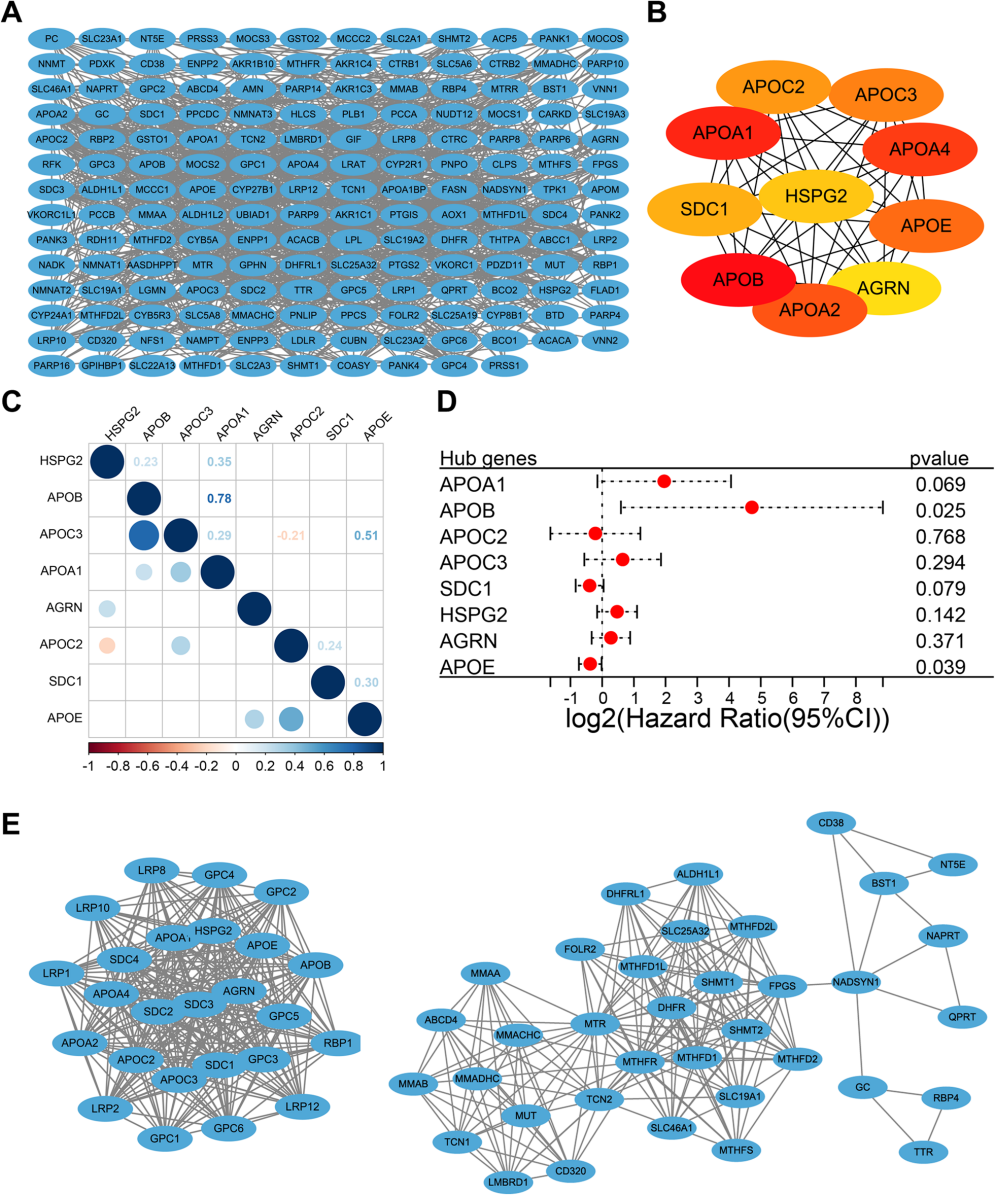
PPI network and hub genes in the vitamin & cofactor metabolic pathway. **A** PPI network of vitamin & cofactor metabolic pathway genes according to the STRING database. **B** The top 10 hub genes of vitamin & cofactor metabolic pathway genes. **C** Correlations among eight matched hub genes in the TARGET cohort. Red representing negative correlations and blue representing positive correlations. Blank represents a correlation P-value > 0.05. **D** Univariate Cox regression analysis of overall survival for eight hub genes. **E** The two gene modules identified from the PPI network

Given the importance of the vitamin & cofactor metabolic pathway, we constructed a PPI network of pathway genes and found extensive interactions (Fig. 2A). In addition, the top 10 hub genes in the vitamin & cofactor metabolic pathway were identified according to the PPI network (Fig. 2B). Notably, most of the hub genes belongs to the apolipoprotein (APO) family, indicating the central role of APO family genes in vitamin & cofactor metabolism. Eight hub genes were subsequently matched in the RNA-Seq data of the TARGET cohort, and correlation analysis showed some positive correlations among the APO family genes in the eight hub genes (Fig. 2C). Univariate Cox regression analysis showed that APOB and APOE were significantly associated with OS in patients with osteosarcoma (Fig. 2D). Furthermore, two gene modules were also identified from the PPI network, one of which contains many APO family genes (Fig. 2E). Furthermore, hub genes were identified in three other prognostic-related metabolic super-pathways (Additional file 1: Figure S8-S10). Among them, the top 10 hub genes in the carbohydrate metabolism pathway are not associated with prognosis. The top 10 hub genes in the energy metabolism pathway are mainly composed of the G protein family, and GNG4 and GNG10 have prognostic significance. The top 10 hub genes in the lipid metabolism pathway are mainly composed of the mediator complex family, and only CD36 among the top 10 genes has prognostic significance. Moreover, we have also constructed interaction networks and identified hub genes in three additional non-prognostic metabolic super-pathways (Additional file 1: Figure S11-S13).

### Identification of metabolic pathway-related clusters and the relationship between clusters and TIME in osteosarcoma

**Fig. 3:**
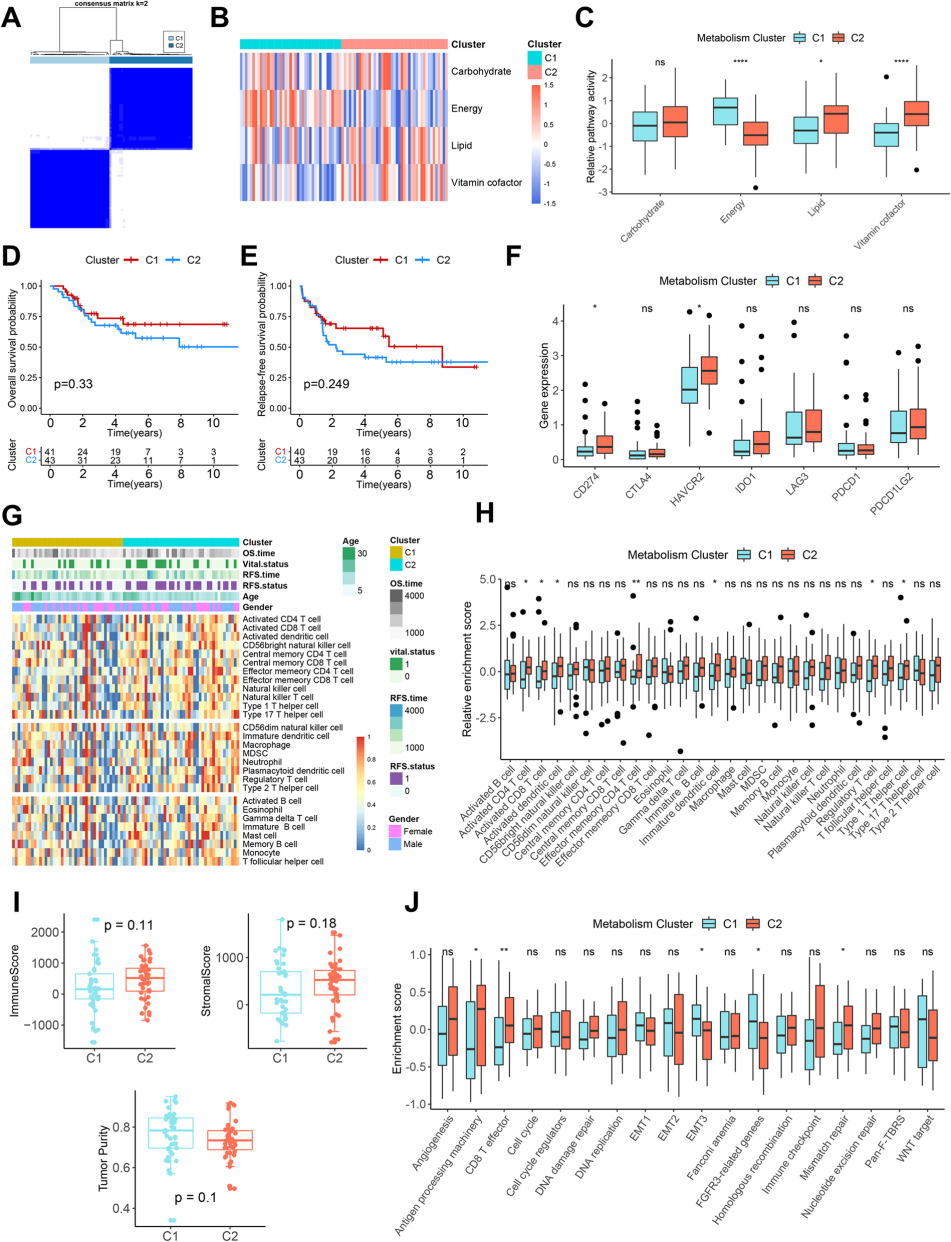
Metabolic pathway-related clusters and the relationship between clusters and TIME in osteosarcoma. **A** Consensus heatmap based on four key metabolic pathways in the TARGET cohort. **B** The heatmap of four key metabolic pathways between C1 and C2. **C** Differences of four key metabolic pathways between C1 and C2. **D**, **E** Kaplan–Meier curves depict the OS (**D**) and RFS (**E**) difference between C1 and C2. Red representing the C1 patients and blue representing the C2 patients. **F** Differences of immune checkpoint genes expression between C1 and C2.** G** The heatmap of 28 immune cells between the two clusters and the correlations of the clusters and clinical parameters. **H** Differences of the abundance of 28 immune cells between C1 and C2.** I** Differences of ImmuneScore, StromalScore and tumor purity between C1 and C2. **J** Differences of core biological pathway activity between C1 and C2. *P < 0.05, **P < 0.01, ****P < 0.0001

To systematically assess the metabolic patterns of osteosarcoma, the four key metabolic pathways were analyzed using unsupervised clustering. As shown in Fig. 3A, osteosarcoma samples can be clearly divided into two distinct metabolic pathway-related clusters (C1 and C2). C1 has a higher level of energy metabolism compared to C2, while C2 has a higher lipid and vitamin & cofactor metabolism (Fig. 3B, C). Survival analysis showed that C1 patients had relatively better long-term OS and RFS, but it did not reach statistical significance, which could be attributed to the limitation in sample size (Fig. 3D, E).

We also explored the relationship between metabolic clusters and osteosarcoma TIME. Immune checkpoint analysis showed that C2 had higher CD274 (PD-L1) and HAVCR2 expression than C1 (Fig. 3F), suggesting higher immunosuppression in C2. Immune cell infiltration analysis showed that C2 had a higher infiltration of activated CD4 and CD8 T cells, as well as a higher infiltration of regulatory T cells that exerted immunosuppressive effects (Fig. 3G, H). However, there was no significant difference in the overall level of immune infiltration between C1 and C2 samples (Fig. 3I). Core biological pathway analysis revealed that C2 had higher levels of antigen processing, CD8 T effector and mismatch repair, and C1 had higher levels of EMT and fibroblast growth factor receptor 3 (FGFR3)-related genes (Fig. 3J). It is noteworthy that we also identified two clusters based on seven metabolic pathways (7MC1 and 7MC2). However, there were no obvious differences observed in terms of prognosis, immune checkpoint expression, immune infiltration, and core biological pathways between 7MC1 and 7MC2 (Additional file 1: Figure S14).

### Identification of MRG-related gene clusters

**Fig. 4:**
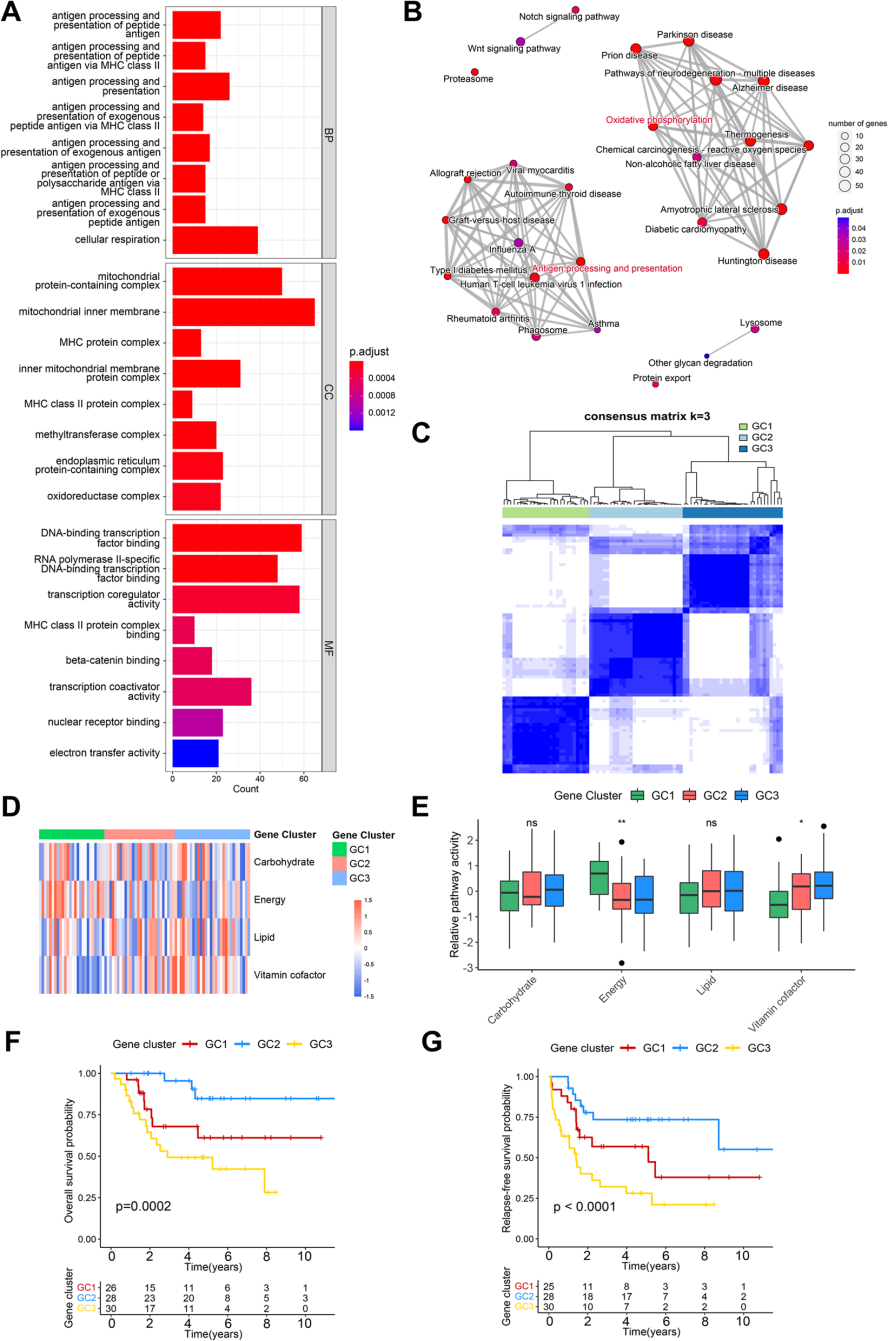
Enrichment analysis of MRGs and identification of MRG-related gene clusters. **A** The top eight enriched terms in GO enrichment analysis of MRGs. **B** The KEGG pathway analysis networks of MRGs. **C** Consensus heatmap based on MRGs in the TARGET cohort. **D** The heatmap of four key metabolic pathways among GC1-3. **E** Differences of four key metabolic pathways among GC1-3. **F**, **G** Kaplan–Meier curves depict the OS (**F**) and RFS (**G**) difference among GC1-3. Red representing the GC1 patients, blue representing the GC2 patients, and yellow representing the GC3 patients. *P < 0.05, **P < 0.01

To further characterize and understand the biological features of metabolic pathway-related clusters and to find a more effective classification, we identified 1218 differentially expressed MRGs between C1 and C2. Not surprisingly, GO enrichment analysis showed that MRGs were mainly enriched in biological processes related to antigen presentation and cellular respiration as well as mitochondria-related cellular components (Fig. 4A). KEGG pathway analysis also showed that MRGs were enriched in pathways represented by antigen processing and oxidative phosphorylation (Fig. 4B). Then 114 representative prognosis-related MRGs were identified by Cox regression analysis (Additional file 4: Table S3). Based on these representative MRGs, osteosarcoma patients were divided into 3 distinct patient groups, termed MRG-associated gene clusters 1–3 (GC1-3, Fig. 4C). GC1 had the highest level of energy metabolism among the three clusters, while GC2 and 3 had higher level of vitamin & cofactor metabolism (Fig. 4D, E). Survival analysis showed significant prognostic differences among gene clusters, with GC2 having the best OS (*p* = 0.0002) and the best RFS (*p* < 0.0001), while GC3 had the worst (Fig. 4F, G).

### The relationship between MRG-related gene clusters and TIME in osteosarcoma

**Fig. 5:**
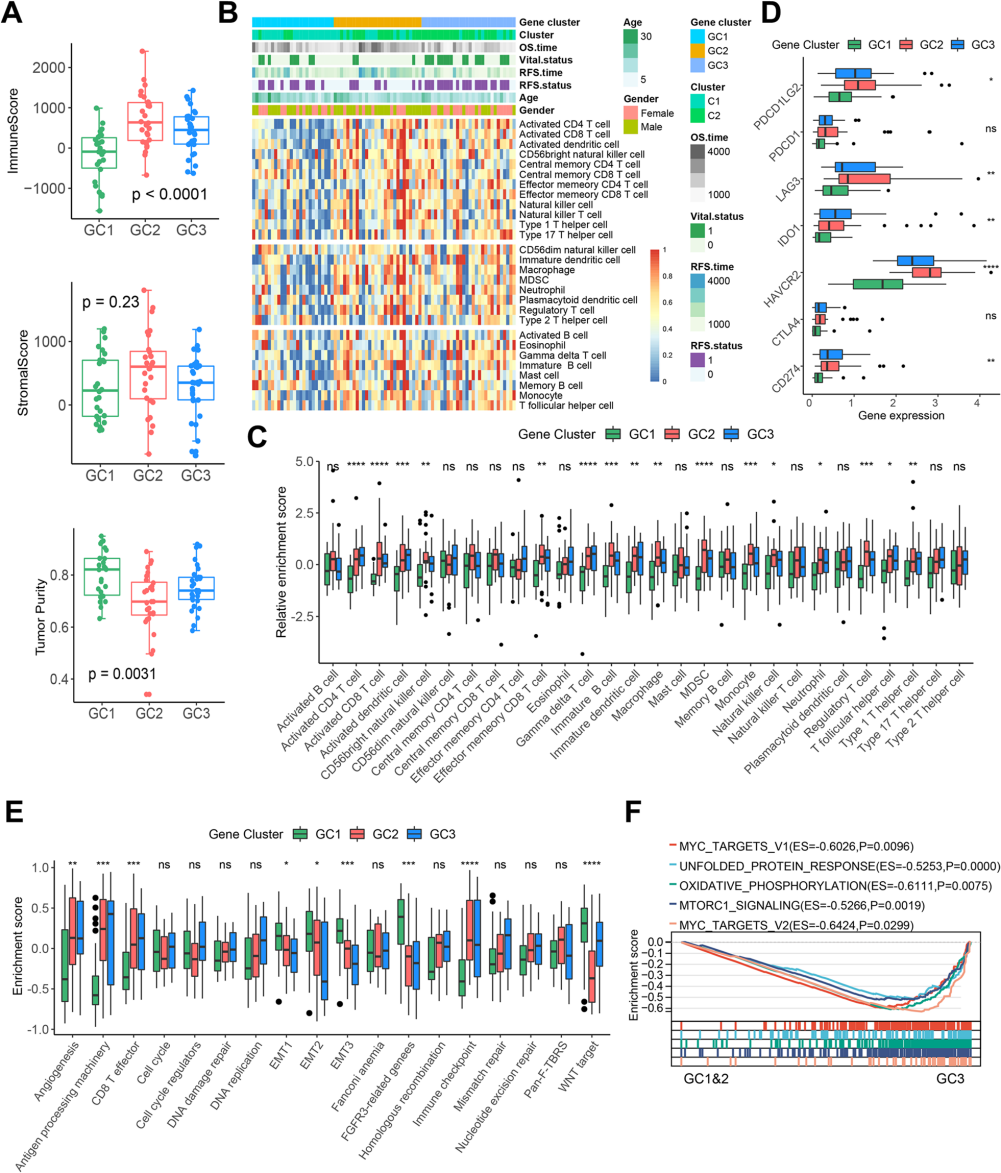
The relationship between MRG-related gene clusters and TIME in osteosarcoma. **A** Differences of ImmuneScore, StromalScore and tumor purity among GC1-3. **B** The heatmap of 28 immune cells among the three gene clusters and the correlations of the gene clusters and clinical parameters. **C** Differences of the abundance of 28 immune cells among GC1-3.** D** Differences of immune checkpoint genes expression among GC1-3. **E** Differences of core biological pathway activity among GC1-3. **F** GSEA enrichment plot based on the HALLMARK gene set showing the relatively significantly enriched pathways in GC3 patients. *P < 0.05, **P < 0.01, ***P < 0.001, ****P < 0.0001

To understand whether significant prognostic differences among gene clusters were associated with TIME, we first assessed the overall immune infiltration differences across GC1-3. As shown in Fig. 5A, GC2 had the highest ImmuneScore and the lowest tumor purity, representing a better immune response, while there was no significant difference in StromalScore among gene clusters. More detailed analysis of immune cell infiltration showed that GC2 and GC3 had higher infiltration of immune activating and immunosuppressive cells (Fig. 5B, C). In addition, GC2 and GC3 also had higher expression of immune checkpoint genes (Fig. 5D). Although in the core biological pathway analysis both GC2 and GC3 had higher activity of immune-related pathways such as antigen processing, immune checkpoint and CD8 T effector and lower EMT and FGFR3-related genes. GC2 had significantly lower Wnt signaling pathway activity than GC3 (Fig. 5E), which may be one of the reasons of the prognostic differences between the two. Further GSEA analysis showed that GC3 had the worst prognosis despite high immune infiltration probably due to the highly activated MYC and MTOR pathways (Fig. 5F).

### Construction of a metabolism-related risk model and the relationship between the risk model and TIME

**Fig. 6:**
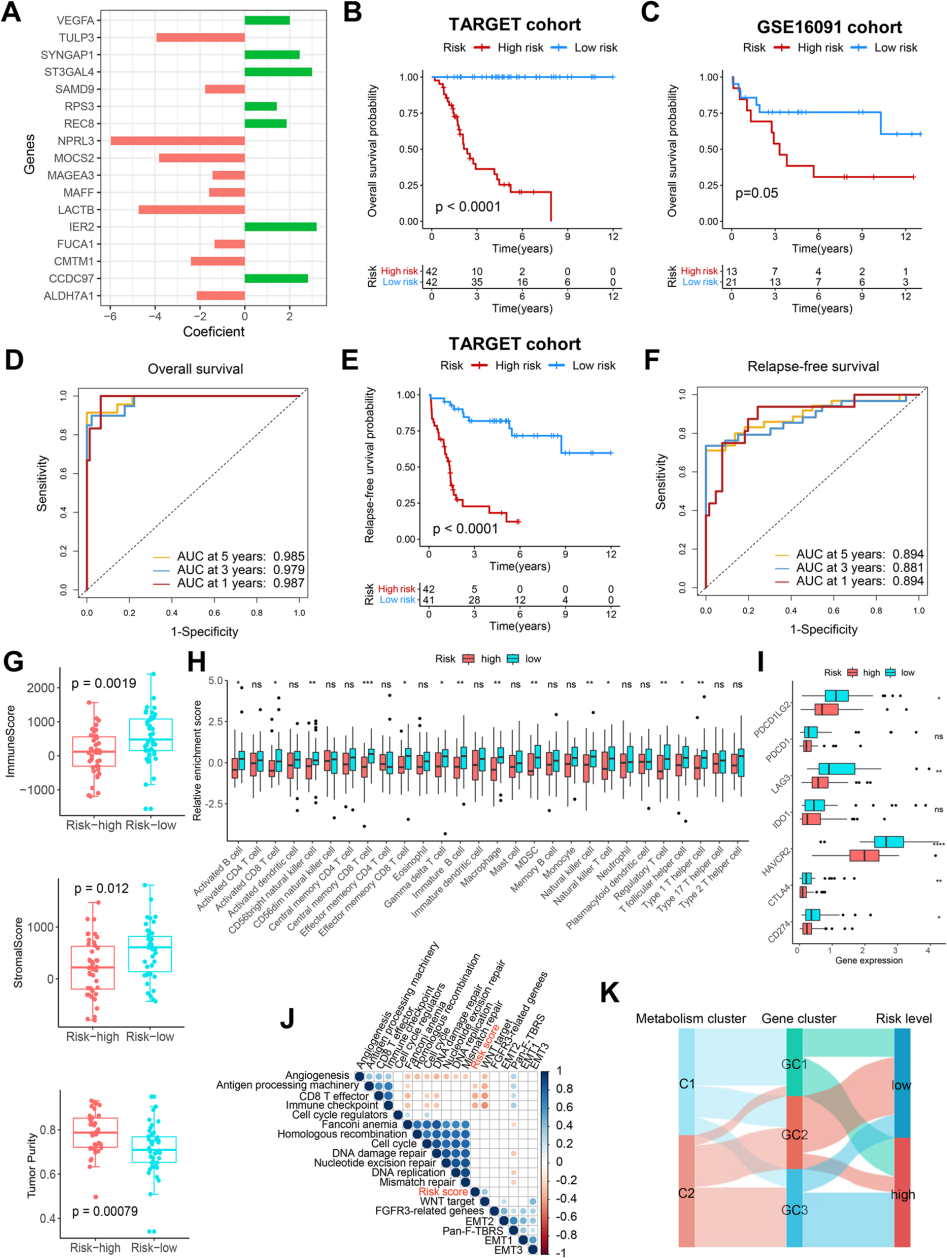
Construction of a metabolism-related risk model and the relationship between the risk model and TIME. **A** Coefficients for the 17 genes in the risk model. **B** Kaplan–Meier curve depicts the OS difference between high-risk and low-risk groups in the TARGET cohort. Red representing the high-risk group and blue representing the low-risk group. **C** Kaplan–Meier curve depicts the OS difference between high-risk and low-risk groups in the GSE16091 cohort. Red representing the high-risk group and blue representing the low-risk group.** D** ROC curve analysis of the risk score for OS in the TARGET cohort.** E** Kaplan–Meier curve depicts the RFS difference between high-risk and low-risk groups in the TARGET cohort. Red representing the high-risk group and blue representing the low-risk group.** F** ROC curve analysis of the risk score for RFS in the TARGET cohort. **G** Differences of ImmuneScore, StromalScore and tumor purity between high and low risk groups.** H** Differences of the abundance of 28 immune cells between high and low risk groups.** I** Differences of immune checkpoint genes expression between high and low risk groups.** J** Differences of core biological pathway activity between high and low risk groups. **K** Alluvial diagram of metabolism clusters, MRG-related gene clusters, and risk levels. *P < 0.05, **P < 0.01, ***P < 0.001, ****P < 0.0001

To further construct a metabolism-related risk prediction tool, 114 prognosis-related MRGs were first downscaled and 25 hub prognosis MRGs were identified using LASSO regression analysis (Additional file 1: Figure S15). The optimal risk model containing 17 core MRGs was subsequently constructed by stepwise multivariate regression analysis. There are some correlations among the expression of the 17 MRGs (Additional file 1: Figure S16). Figure 6A illustrates the coefficients of each MRGs in the risk model. After dividing the TARGET cohort into two groups by the median risk score, it was seen that patients with osteosarcoma in the low-risk group had significantly better OS (Fig. 6B, p < 0.0001). Using the GSE16091 cohort as an independent validation cohort, although only 15 risk MRGs could be matched, patients with low risk score in this cohort still had a better prognosis than patients with high risk scores (Fig. 6C, p = 0.05). The ROC curve showed that the risk score was a good predictor of OS in the TARGET cohort, with AUCs of 0.987, 0.979, and 0.985 at 1, 3, and 5 years, respectively (Fig. 6D). In addition, patients with high risk scores had significantly worse RFS (Fig. 6E, p < 0.0001), and the risk score also had good efficiency in predicting RFS (Fig. 6F).

To understand the differences in TIME across risk groups, ESTIMATE analysis was performed. It found that low-risk patients had higher ImmuneScore and StromalScore and lower tumor purity than high-risk patients (Fig. 6G), implying that low-risk patients had higher overall immune and stromal infiltration. The heatmap demonstrated the relationship between risk score and immune cell infiltration and clinical parameters such as survival status (Additional file 1: Figure S17). As shown in Fig. 6H, high-risk patients had significantly lower levels of immune cell infiltration, such as activated CD8 T cells, than low-risk patients. In addition, low-risk patients also had higher immune checkpoint gene expression (Fig. 6I). In the correlation analysis, consistent with these findings, the risk score was negatively correlated with the level of immune cells infiltration and the expression of immune checkpoint genes (Additional file 1: Figure S18A-C). In the core biological pathway analysis, the risk score potentially exhibited a negative correlation with antigen processing, CD8 T effector, and immune checkpoint (Fig. 6J). Differential analysis revealed that patients with high-risk scores had lower enrichment levels of CD8 T effector and immune checkpoints (Additional file 1: Figure S18D). The alluvial diagram in Fig. 6K demonstrated the relationship among metabolism clusters, MRG-related gene clusters, and risk levels. GC1 patients were mainly from C1 and GC3 patients were mainly from C2. In addition, most GC2 patients were assigned to the low-risk group and most GC3 patients were assigned to the high-risk group.

### Immunotherapy response and drug sensitivity analysis

**Fig. 7:**
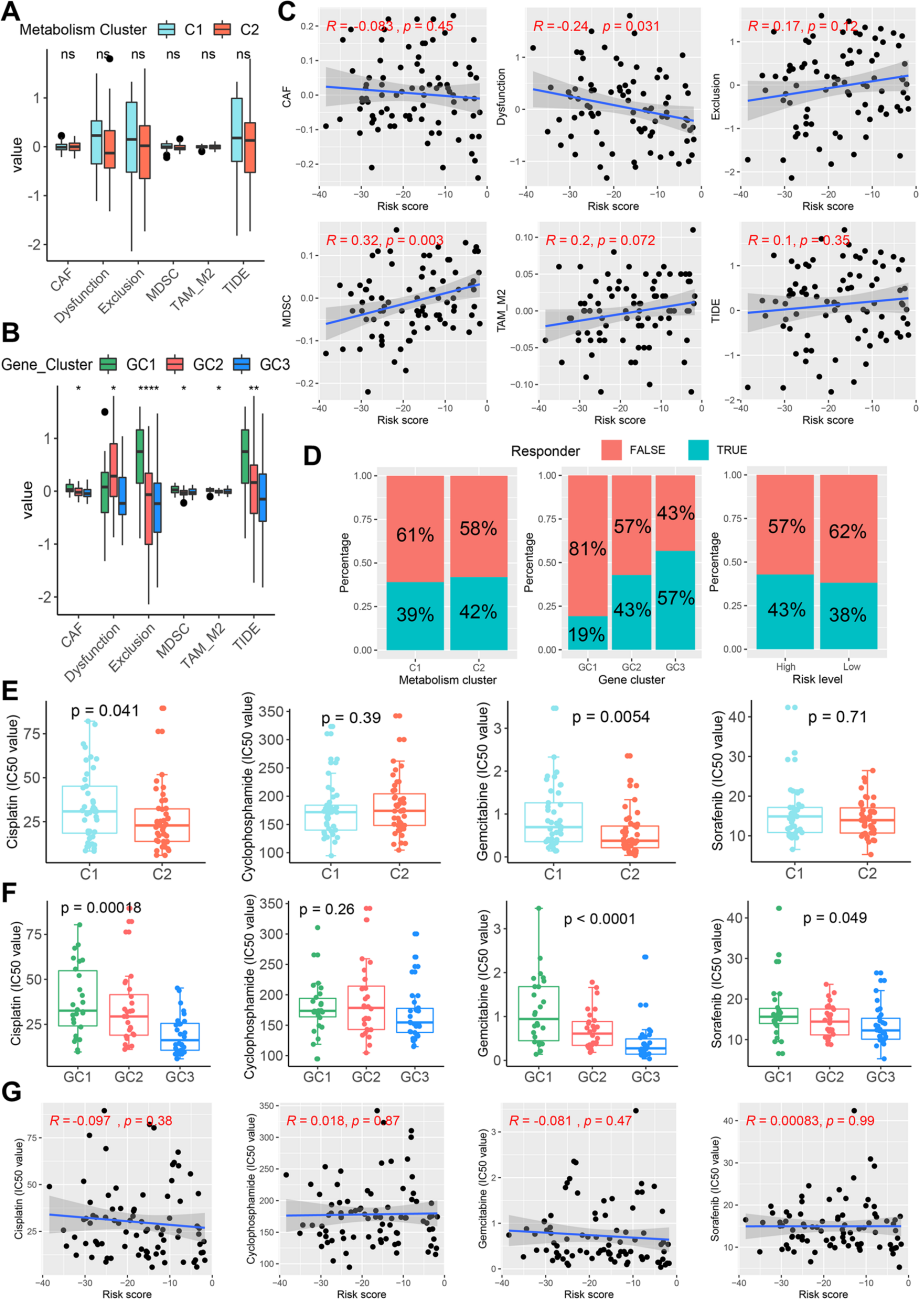
Immunotherapy response and drug sensitivity analysis. **A** Differences of TIDE-related scores between C1 and C2. **B** Differences of TIDE-related scores among GC1-3.** C** Correlations of the risk score with TIDE-related scores. **D** Rate of predicted clinical response to ICB immunotherapy in different clusters and risk levels.** E** Differences in IC50 values of cisplatin, cyclophosphamide, gemcitabine and sorafenib between C1 and C2.** F** Differences in IC50 values of cisplatin, cyclophosphamide, gemcitabine and sorafenib among GC1-3. **G** Correlations of the risk score with IC50 values of cisplatin, cyclophosphamide, gemcitabine and sorafenib. *P < 0.05, **P < 0.01, ****P < 0.0001

It is well known that higher TIDE score is associated with lower response to ICB treatment [39].The TIDE score helps in the clinical selection of patients who may be suitable for ICB treatment and in identifying responders. This study explored the relationship between metabolism clusters, MRG-related gene clusters, and risk score with ICB treatment response by TIDE score. There is no difference in TIDE score between the different metabolism clusters (Fig. 7A). Among the different MRG-related gene clusters, GC3 had the lowest CTL dysfunction, exclusion, and TIDE scores (Fig. 7B), indicating a good response to ICB treatment. The risk score is potentially negatively correlated with the CTL dysfunction score, but potentially positively correlated with the MDSC score (Fig. 7C). Consistent with these results, there was no significant difference in ICB response between C1 and C2 and between high and low risk groups, but GC3 had the highest proportion of ICB responders (Fig. 7D), suggesting that the MRG-related gene cluster may be a good predictor of ICB response.

Three guideline-based used chemotherapeutic agents (cisplatin, cyclophosphamide and gemcitabine) for osteosarcoma and one targeted agent (sorafenib) with clinical application value were retrieved from the GDSC database. Then, the relationship of these drugs with different clusters as well as the risk score was explored. As shown in Fig. 7E, C2 patients had lower half-maximal inhibitory concentration (IC50) values for cisplatin and gemcitabine than C1 patients, indicating that C2 patients were more sensitive to cisplatin and gemcitabine. Among the MRG-related gene clusters, the IC50 values of cisplatin, gemcitabine, and sorafenib were sequentially lower in GC1-GC3, indicating the different sensitivity of the three clusters to these three drugs (Fig. 7F). Although the risk score did not correlate with IC50 values for these drugs (Fig. 7G), we screened for 18 drugs that were significantly associated with the risk score (Additional file 5: Table S4). Therefore, the risk score may serve as the predictor of sensitivity to these drugs.

### ST3GAL4 is highly expressed in malignant cells and is closely associated with the TIME of osteosarcoma

**Fig. 8:**
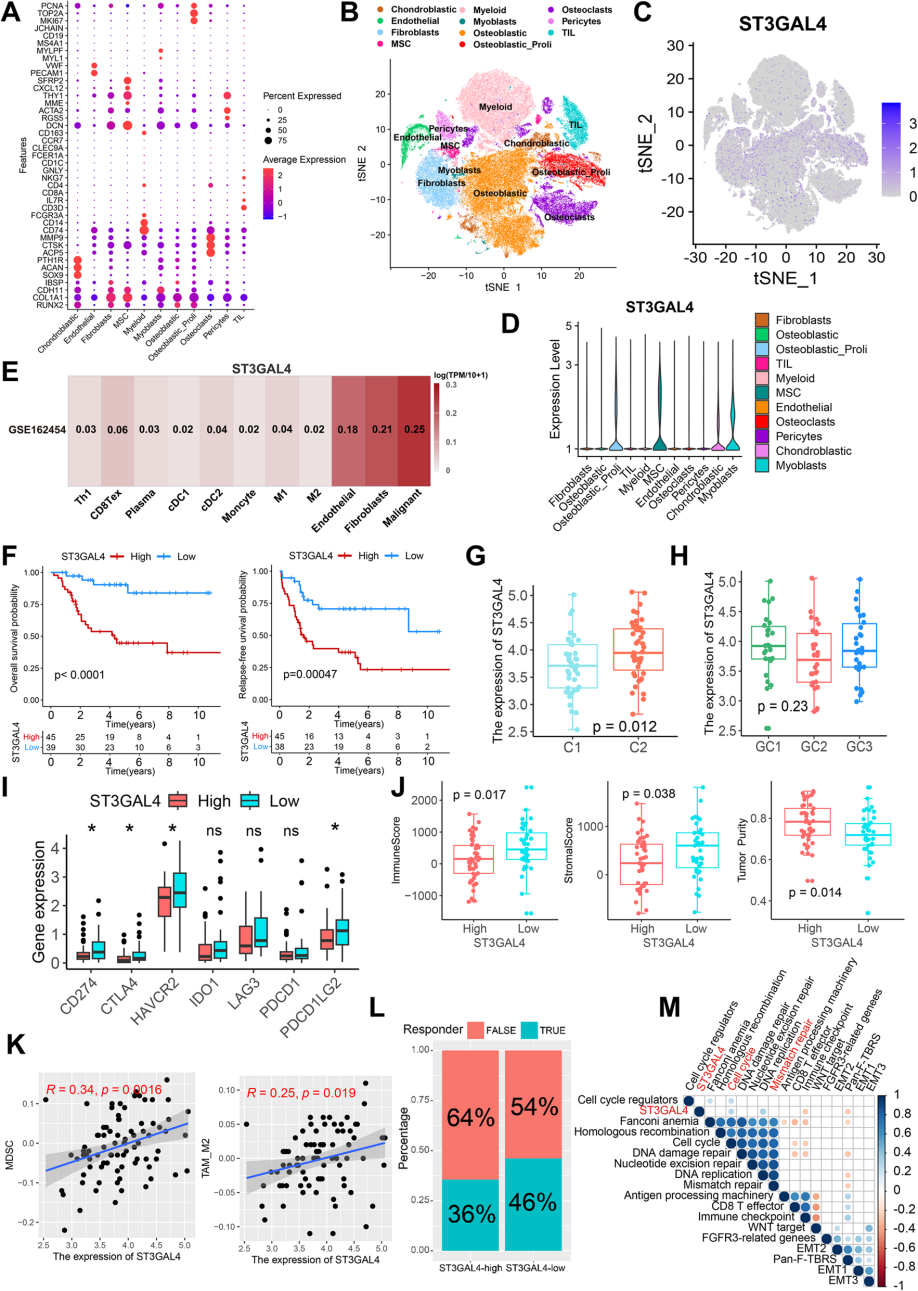
ST3GAL4 is highly expressed in malignant cells and is closely associated with the TIME of osteosarcoma. **A** The dot plot shows the expression of 41 characteristic genes in 11 cell clusters. The size of the dots indicates the proportion of cells expressing a specific marker, and the color indicates the average expression level of the markers. MSC, mesenchymal stem cell; TIL, tumor-infiltrating lymphocyte. **B** The t-SNE plot of the 11 main cell types in osteosarcoma. **C** Feature plot for ST3GAL4. The color legend shows the normalized expression levels of the genes. **D** Violin plot showing the normalized expression levels of ST3GAL4 across the 11 cell types. **E** Expression of ST3GAL4 among different cell types in the GSE162454 cohort. The color indicates the average expression level of ST3GAL4. **F** Kaplan–Meier curves depict the OS and RFS difference between high-ST3GAL4 and low-ST3GAL4 groups in the TARGET cohort. Red representing the high-ST3GAL4 group and blue representing the low-ST3GAL4 group. **G** Differences of the expression of ST3GAL4 between C1 and C2. **H** Differences of the expression of ST3GAL4 among GC1-3.** I** Differences of immune checkpoint genes expression between high-ST3GAL4 and low-ST3GAL4 groups. *P < 0.05.** J** Differences of ImmuneScore, StromalScore and tumor purity between high-ST3GAL4 and low-ST3GAL4 groups. **K** Correlations of the expression of ST3GAL4 with MDSC score and TAM-M2 score. **L** Rate of predicted clinical response to ICB immunotherapy in high-ST3GAL4 and low-ST3GAL4 groups. **M** Correlations between the expression of ST3GAL4 and known core biological pathway scores. Correlation coefficients are calculated by Spearman’s correlation analysis, with red representing negative correlations and blue representing positive correlations. Blank represents a correlation P-value > 0.05

In the above results we identified 17 core MRGs to construct a risk model. The advent of scRNA-seq has enabled researchers to investigate the activity of genes across diverse cell types. The activation of genes in malignant cells can significantly impact their biological behavior, consequently influencing tumor progression. To delve deeper into the expression patterns of the 17 core MRGs across distinct cell types, we initially identified 11 major cell types using characteristic gene expression in the scRNA-seq cohort GSE152048 (Fig. 8A, B). Subsequently, ST3GAL4 was found to not only have a high positive coefficient in the risk model, but also to be predominantly expressed in malignant cells (osteoblastic and chondroblastic osteosarcoma cells) compared to other core MRGs (Fig. 8C, Additional file 1: Figure S19 A, C). Notably, the violin plot showed that ST3GAL4, rather than other MRGs, was specifically highly expressed in proliferating osteoblastic osteosarcoma cells (Fig. 8D, Additional file 1: Figure S19 B, D), suggesting the potentially important role of ST3GAL4 in the proliferation of osteosarcoma cells. Importantly, it was also verified in the scRNA-seq cohort GSE162454 that ST3GAL4 was predominantly expressed in malignant cells (Fig. 8E).

Further, osteosarcoma patients with high ST3GAL4 were found to have significantly worse OS and RFS (Fig. 8F, all *p* < 0.001). C2 samples had significantly higher ST3GAL4 expression compared to C1 samples (Fig. 8G), and, although not statistically significant, GC2 samples had relatively lower ST3GAL4 expression compared to GC1 and GC3 samples (Fig. 8H). Immune checkpoint analysis revealed that samples with high ST3GAL4 had significantly lower expression of CD274, cytotoxic T-lymphocyte associated protein 4 (CTLA4), HAVCR2 and programmed cell death 1 ligand 2 (PDCD1LG2) (Fig. 8I). Samples with high expression of ST3GAL4 also had lower ImmuneScore, StromalScore and higher tumor purity (Fig. 8J). Analysis based on the TIDE algorithm revealed that ST3GAL4 expression was positively correlated with MDSC score and TAM-M2 score (Fig. 8K), but not correlated with CAF score, TIDE score, and dysfunction and exclusion of CTLs (Additional file 1: Figure S20). Taken together, samples with high ST3GAL4 may have difficulty responding to ICB treatment. The response prediction based on the TIDE algorithm also demonstrated that patients with high ST3GAL4 had a relatively low ICB response rate (Fig. 8L). However, there was no significant associations between ST3GAL4 and sensitivities to cisplatin, cyclophosphamide, gemcitabine and sorafenib (Additional file 1: Figure S21). ST3GAL4 expression was potentially positively correlated with cell cycle and mismatch repair and potentially negatively correlated with immune checkpoint and Pan-F-TBRS (Fig. 8M).

The ST3GAL family consists of six members (ST3GAL1-6), and it is necessary to further analyze the other members of this family. ScRNA-seq analysis showed that other ST3GAL members were not specifically highly expressed in proliferating malignant cells (Additional file 1: Figure S22A, B). In addition, survival analysis showed that only ST3GAL1 was associated with shorter OS and RFS in osteosarcoma, but its prognostic value was not as significant as ST3GAL4 (Additional file 1: Figure S22C-G). Immune-related analysis found that only ST3GAL2 was associated with overall immune infiltration in osteosarcoma, but immune checkpoint analysis failed to find a widespread correlation (Additional file 1: Figure S22H, I). Furthermore, it was found that ST3GAL5 and ST3GAL6 were negatively correlated with TIDE score, MDSC score, CAF score, exclusion of CTLs, and some core biological pathways including EMT (Additional file 1: Figure S22J, K). In summary, only ST3GAL4 in the ST3GAL family is associated with both the prognosis and immune characteristics of osteosarcoma.

### ST3GAL4 is a potential prognostic marker and associated with tumor progression, glycolysis and the M2 polarization of macrophages in osteosarcoma

**Fig. 9:**
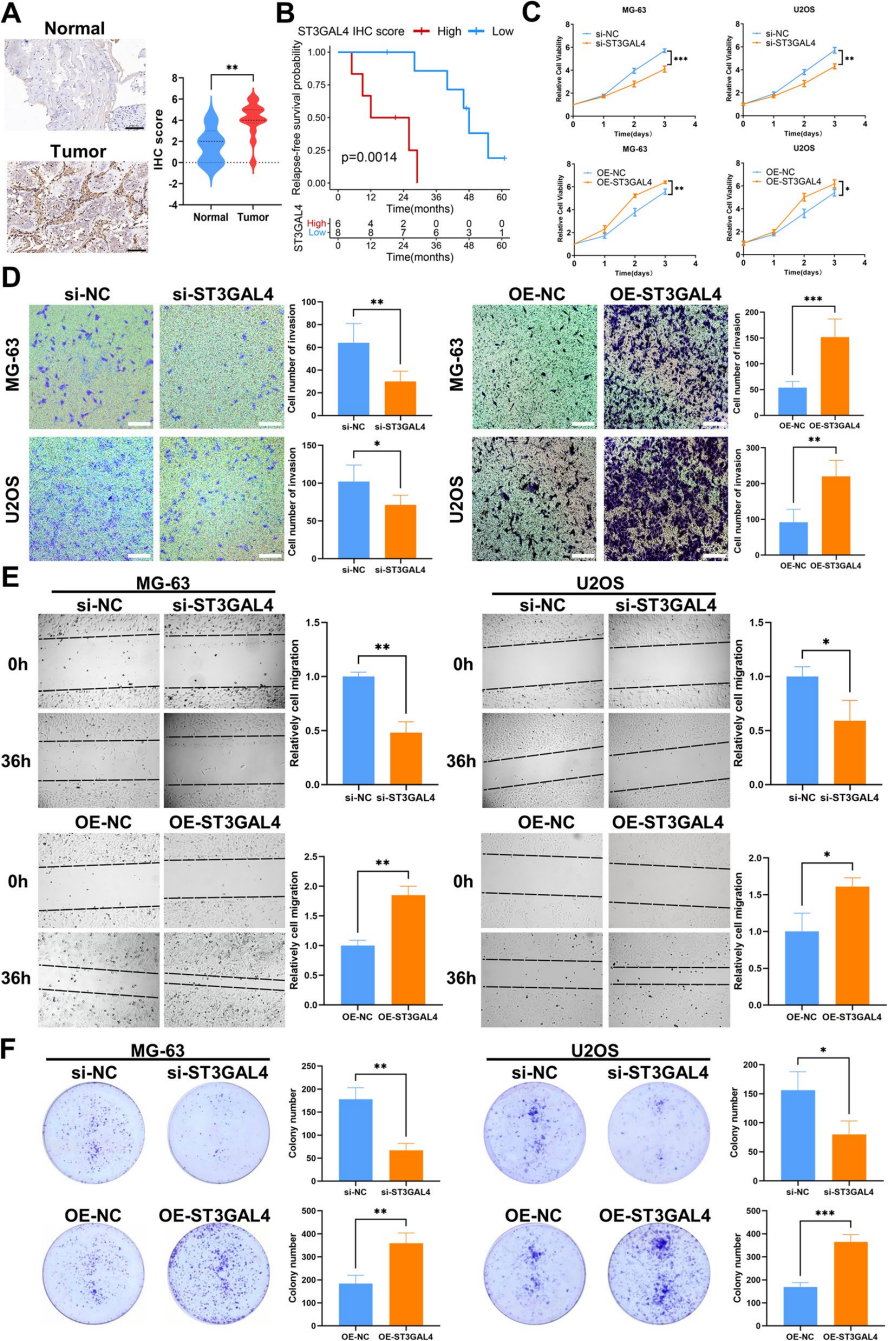
Protein expression of ST3GAL4 in osteosarcoma tissues and its effects on proliferation, invasion and migration of osteosarcoma cells. **A** IHC staining images of ST3GAL4 in osteosarcoma tissues (#6, n = 14) and corresponding normal tissues (#2, n = 5). The IHC scores indicated that the protein expression of ST3GAL4 was higher in tumor tissues. **B** Kaplan–Meier curve depicts the RFS difference between high and low ST3GAL4 protein group in the Xiangya cohort. Red representing the high ST3GAL4 protein group and blue representing the low ST3GAL4 protein group. **C** Folded line plots showing the effect of ST3GAL4 knockdown and overexpression on the proliferation of MG-63 and U2OS cells. The blue line represents the control group and the yellow line represents the knockdown/overexpression group. **D** Transwell chamber experiments showing the effect of ST3GAL4 knockdown and overexpression on the invasion of MG-63 and U2OS cells. Scale bar: 100 μm. **E** Scratch assays showing the effect of ST3GAL4 knockdown and overexpression on the migration of MG-63 and U2OS cells. **F** Colony formation assays showing the effect of ST3GAL4 knockdown and overexpression on the ability of colony formation of MG-63 and U2OS cells. Data are represented as mean ± SEM. *P < 0.05, **P < 0.01, ***P < 0.001

To verify the clinical application, IHC staining was performed on osteosarcoma and normal tissue samples. The protein expression of ST3GAL4 was found to be significantly higher in tumor tissue than in normal tissue (Fig. 9A). Survival analysis indicated that patients with high ST3GAL4 protein expression had shorter RFS (Fig. 9B, p = 0.0014). We knocked down and overexpressed ST3GAL4 in the osteosarcoma cell lines MG-63 and U2OS to explore its effect on the malignant phenotype of osteosarcoma cells. After the knockdown of ST3GAL4, the proliferation, invasion, migration and the ability of colony formation of MG-63 and U2OS were all inhibited (Fig. 9C–F). After overexpressing ST3GAL4, the malignant phenotypes mentioned above were all enhanced.

**Fig. 10:**
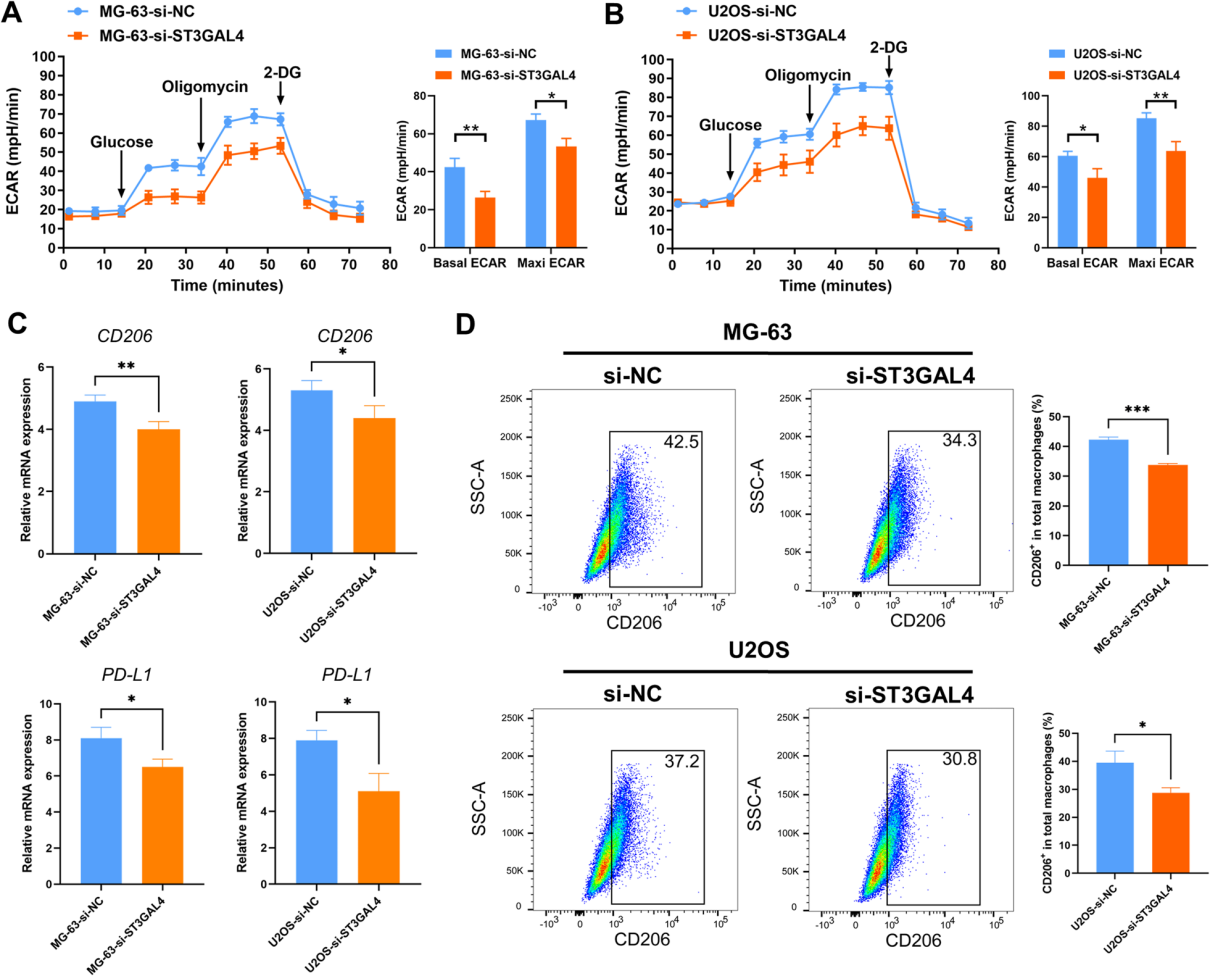
ST3GAL4 regulates the glycolysis of tumor cells and the M2 polarization of macrophages in osteosarcoma. **A, B** Seahorse assays indicated that the knock down of ST3GAL4 inhibited glycolysis in osteosarcoma cells. Left, representative curve; Right, quantification of basal ECAR and maxi ECAR. ECAR, extracellular acidification rate. **C** RT-qPCR analysis is shown for PD-L1 and M2 marker CD206 in macrophages. **D** Flow cytometry analysis is shown for expression of CD206 in macrophages cultured with si-NC or si-ST3GAL4 tumor cells. Shown are representative plots and quantification of the percentage of CD206 positive cells in total macrophages

Although no correlation was found between ST3GAL4 and the four metabolic super-pathways (Additional file 1: Figure S23), based on previous studies [44–48], we speculate that ST3GAL4 may have a potential association with glycolysis. To further explore the relationship between ST3GAL4 and glycolysis, seahorse assay was conducted. As expected, the knock down of ST3GAL4 reduced the basal glycolysis level and maximal glycolysis level in osteosarcoma cells (Fig. 10A, B). Furthermore, through the co-culture system, we explored the impact of ST3GAL4 on macrophage polarization. RT-PCR analysis showed that the knock down of ST3GAL4 significantly decreased the expression of the M2 macrophage marker CD206 (Fig. 10C). Interestingly, the expression of PD-L1 in macrophages was also reduced in the ST3GAL4 knockdown group (Fig. 10C). Flow cytometry analysis further confirmed a lower proportion of M2 macrophages in the ST3GAL4 knockdown group compared to the control group, confirming the regulation of ST3GAL4 on macrophage polarization in osteosarcoma (Fig. 10D).

## Discussion

Metabolic reprogramming is considered to be one of the hallmarks of cancer [2, 49]. The metabolic activity of cancer is extremely complex and needs to be systematically characterized. However, several previous studies have demonstrated considerable heterogeneity in the expression of genes involved in various metabolic pathways [50–54], and thus, metabolic gene expression alone cannot accurately reflect the metabolic changes in tumors. Based on studies with parallel metabolomics data as well as transcriptomics data, Peng et al. proved that metabolic pathway-based expression patterns reflect the true metabolic status well [26]. Therefore, the investigation of osteosarcoma metabolism from metabolic pathways is equally promising. Current studies on the metabolic profile of osteosarcoma tend to focus only on a specific metabolic pathway [23, 24, 55], and the authors are not aware that any studies have yet examined the impact of different metabolic pathways on osteosarcoma from a holistic perspective. In addition, it is well known that metabolic activity greatly influences the formation of TIME [15–17], therefore it is necessary to further reveal the relationship between metabolism and TIME in osteosarcoma.

In this study, we first explored the impact of enrichment levels of the seven most prominent metabolic super-pathways on the prognosis of osteosarcoma. Unexpectedly, four of the seven metabolic super-pathways (carbohydrate, lipid, energy, and vitamin & cofactor) were all associated with better OS in osteosarcoma. This appears to be a departure from previous knowledge that cancer cells have an increased need for glucose and energy uptake [56]. However, in agreement with our study, Peng et al. found that lipid metabolism was associated with a better prognosis for a variety of tumors in a pan-cancer analysis, and energy metabolism showed a heterogeneous prognostic correlation [26]. Notably, they found that carbohydrate and vitamin & cofactor metabolism were associated with worse prognosis in tumors, which is different to our results in osteosarcoma. They are highly heterogeneous tumors containing multiple subtypes including osteoblastic and chondroblastic osteosarcoma [57]. The exact characteristics of osteosarcoma metabolism remain to be elucidated, which may result in a different metabolic pattern from other tumors as well as clinical relevance. It should not be overlooked that a large number of previous studies have focused on carbohydrate metabolism in osteosarcoma [57–60]. In this study, the most significant difference was found between OS of osteosarcoma with different levels of vitamin & cofactor metabolism, implying that vitamin & cofactor metabolism may largely influence the prognosis of osteosarcoma. Importantly, vitamin & cofactor metabolism was strongly correlated with immune and stromal cell infiltration in the TIME of osteosarcoma, and carbohydrate and vitamin & cofactor metabolism were also correlated with infiltration levels of various antitumor immune cells such as effector memory CD8 T cells. Previous studies have demonstrated that higher immune cell infiltration in osteosarcoma is associated with better prognosis [9], which may partially explain why osteosarcoma patients with high levels of carbohydrate and vitamin & cofactor metabolism have better clinical outcomes. Given the importance of vitamin & cofactor metabolism in osteosarcoma prognosis and TIME, further in-depth study of its mechanism in osteosarcoma and development of therapeutic strategies targeting the vitamin & cofactor metabolism may be promising. Further, we identified the hub genes in the vitamin & cofactor metabolic pathway. Remarkably, most of the hub genes belonged to the APO family. APOs are proteins that bind to lipids (such as cholesterol and triglycerides) in the blood, forming lipoproteins and transporting them through the bloodstream to cells and tissues. Lipoproteins play an important role in vitamin metabolism. For example, APOA is the main component of high-density lipoprotein (HDL) and is directly correlated with the level of vitamin E in the blood, promoting its absorption in the intestines [61]. In colon cancer cells, APOB also participates in the transport of vitamin E [62]. In addition, APOE largely affects the concentration of fat-soluble vitamins in plasma [63]. Recent studies have found that APOC1 promotes osteosarcoma progression through binding to MTCH2 [64], and preoperative APOB/APOA1 has been identified as an independent prognostic factor for osteosarcoma in children and adolescents [65]. In addition, APOD induced the osteoblastic differentiation of the osteosarcoma cell line Sao-2 [66]. This suggests that the APO family may regulate osteosarcoma vitamin & cofactor metabolism and affect the prognosis of osteosarcoma.

Based on the activity levels of the four clinically relevant key metabolic super-pathways, two distinct metabolic pathway-related clusters (C1 and C2) were identified in a cluster analysis. C1 is mainly characterized by energy metabolism, while C2 is characterized by lipid and vitamin metabolism. Although C1 and C2 do have distinct metabolic characteristics, there was no significant difference in survival between them due to the limitation in sample size. It is necessary to explore the prognostic differences between them in larger cohorts in the future. It is noteworthy that C2 patients were more sensitive to cisplatin and gemcitabine. Therefore, this classification may be appropriate to identify osteosarcoma patients with different metabolic profiles and to guide the dosing of cisplatin and gemcitabine. To further explore the clinical significance of the metabolic profile of osteosarcoma, we identified MRGs based on metabolic pathway-related clusters. It should not be overlooked that MRGs were enriched not only in metabolism-related pathways, such as oxidative phosphorylation, but also in immune-related pathways, including antigen processing and presentation. Previous studies have demonstrated that metabolites in TIME affect the differentiation and function of immune cells, thereby modulating the immune response [67, 68]. The results further emphasize the importance of the metabolism in the immune regulation of osteosarcoma. Previous studies have shown that many metabolic genes are also important immunomodulatory genes. For instance, Wolf et al. found that hexokinase, which is involved in the process of glycolysis, is an important regulator of innate immunity [69], demonstrating the overlap between cellular energy metabolism and the immune system. PDCD1 (also known as PD-1), a well-known immune checkpoint gene, has been identified to interact with arginine biosynthesis or fatty acid degradation and elongation [70]. According to the identified MRGs, we defined MRGs-related gene clusters (GC1-3), which may have more important clinical translational implications than metabolic pathway-related clusters. Firstly, the significantly different clinical outcomes among GCs suggest that these GCs reflect essential aspects of tumor development and can be used as potential prognostic predictors. Secondly, different GCs had significantly different TIME, that is, GC2 and GC3 had higher immune infiltration and were more likely to respond to ICB treatment, suggesting that this classification approach may facilitate the development of personalized ICB treatment strategies for osteosarcoma. In addition, differences in sensitivity to cisplatin, gemcitabine and sorafenib among GCs also suggest their potential to guide clinical dosing. From a mechanistic perspective, GC3 with the worst prognosis exhibits higher activity in the MYC and mTOR pathways. MYC is one of the most frequently dysregulated oncogenes known so far, highly expressed in various tumors including osteosarcoma [71]. Its expression promotes tumor progression by providing sufficient energy and metabolic substrates for uncontrolled cell proliferation [72, 73]. The mTOR pathway is also abnormally activated in many cancers, including human osteosarcoma [74]. In osteosarcoma, mTOR promotes cell growth and proliferation, induces cell metastasis, inhibits apoptosis, and suppresses autophagy [74]. Therefore, the activation of the MYC and mTOR pathways may be one of the intrinsic factors contributing to the poor prognosis of GC3. It is worth noting that the Wnt signaling pathway is highly active in both GC1 and GC3. This signaling pathway is associated with tumorigenesis and can regulate the metastasis of osteosarcoma cells through autocrine or paracrine mechanisms, thus reducing patient survival rate [75]. This may be one of the reasons for the poor prognosis of GC3. Admittedly, in GC1, the activation of immune response contributes to a better prognosis even with higher Wnt pathway activity. This reflects the importance of immune response in the survival of GC1.

Furthermore, we downscaled the MRGs by multiple algorithms and identified 17 core MRGs and constructed a risk model. It has shown that risk stratification could significantly improve the treatment outcome of many tumors including osteosarcoma [9, 76, 77]. This risk model has good efficacy and was validated in an independent cohort, suggesting its potential value of clinical application. In addition, the risk model reflects the different immune status for osteosarcoma, whereby patients with higher risk score have lower immune infiltration, which is consistent with previous studies [9]. Remarkably, scRNA-seq-based analysis revealed that ST3GAL4, one of the 17 core MRGs, was highly expressed in proliferating malignant cells. Mechanistically, ST3GAL4 was also associated with cell cycle and mismatch repair, further suggesting that ST3GAL4 influences the development of osteosarcoma. The ST3GAL4 gene encodes the enzyme Galβ1-4GlcNAc α2,3-sialyltransferase. This enzyme is involved in protein glycosylation and the synthesis of the sialyl Lewis x antigen [78]. Previous studies have shown that ST3GAL4 affects several biological behaviors in tumors such as proliferation, invasion, and migration in non-small cell lung cancer and pancreatic cancer cells [42, 79]. The present study demonstrated for the first time that knockdown of ST3GAL4 in cell lines (MG-63 and U2OS) suppressed the malignant phenotype of osteosarcoma. More importantly, we confirmed the clinical feasibility of ST3GAL4 as a prognostic marker in an independent clinical cohort. Although no association was found between ST3GAL4 and four metabolic super-pathways, this study confirmed the involvement of ST3GAL4 in glycolysis in osteosarcoma. Similarly, previous studies indicated that ST6GAL1 regulates glycolysis in ovarian cancer [45]. Liu et al. identified ST3GAL4 as a hypoxia-related gene [47], and it is well-known that a hypoxic microenvironment can induce glycolysis in tumor cells [48]. These findings support our results. Additionally, the hyperactivation of glycolysis in tumors sustains and promotes various malignant behaviors in osteosarcoma cells [80], which may also be a potential mechanism by which ST3GAL4 influences the malignant phenotype of osteosarcoma cells. In addition, it was found that samples with high expression of ST3GAL4 were mainly enriched in cell cycle and DNA repair-related pathways, which may be a potential mechanism by which ST3GAL4 promotes malignant phenotypes (Additional file 1: Figure S24A). We further provided GSEA results of other core MRGs (Additional file 1: Figure S24B) to explore their relationship with tumor-related pathways and suggest potential therapeutic targets.

ST3GAL4 was also associated with the immune response in osteosarcoma and may be an important regulator of the TIME of osteosarcoma. This study confirmed the regulatory effect of ST3GAL4 on macrophage polarization in osteosarcoma using a co-culture system. Studies have demonstrated that lactate, a metabolite generated during the glycolytic process in tumor cells, plays a role in inducing M2 polarization in macrophages, thereby exerting direct immune-suppressive effects [81]. Our findings provide evidence for the involvement of ST3GAL4 in promoting glycolysis, which could partially explain its role in regulating macrophage polarization. A recent study has shown that ST3GAL4 is not only involved in protein glycosylation processes, but also affects the signaling pathways of Siglec-7 and Siglec-9 by promoting the synthesis of ligands in tumor cells, thereby promoting macrophage polarization [82]. This further validates our findings and potentially unveils additional mechanisms through which ST3GAL4 facilitates macrophage M2 polarization. These findings provide support for considering ST3GAL4 as a promising and innovative target for cancer immunotherapy.

However, further investigation is required to further elucidate the role of ST3GAL4 in the TIME of osteosarcoma. Notably, while patients exhibiting high ST3GAL4 expression demonstrated a relatively low response rate to ICB, there was minimal disparity in ICB response rates between the high-risk and risk groups, as well as between subtypes C1 and C2. This is because there is no absolute linear relationship between ST3GAL4 expression and risk score or C1/C2. There are a large number of differentially expressed genes between high and low risk groups or C1 and C2, not just ST3GAL4. Therefore, it is reasonable that there are some differences between ST3GAL4 expression and risk score or C1/C2 in response to ICB, because the population with high/low ST3GAL4 expression does not completely overlap with the population represented by high/low risk group or C1/C2. Overall, our findings support the potential utility of ST3GAL4 as a prognostic marker and a new therapeutic target.

There are still some limitations in this study. Firstly, due to the rarity of osteosarcoma, it is difficult to obtain a large sample cohort to validate the results. Secondly, using an osteosarcoma cohort with parallel metabolomics and transcriptomics data would increase the value of this study. In addition, the effects and mechanisms of vitamin & cofactor metabolism and ST3GAL4 on the TIME of osteosarcoma require further in-depth in vivo and in vitro studies.

## Conclusion

Vitamin & cofactor metabolism plays an important role in the prognosis and TIME of osteosarcoma. MRG-related gene clusters can reflect the immune status of osteosarcoma and facilitate the development of personalized immunotherapy and chemotherapy strategies. The metabolism-related risk model may serve as a useful prognostic predictor. ST3GAL4 plays a critical role in the progression, glycolysis, and TIME of osteosarcoma and affects the prognosis.

### Supplementary Information

**Additional file 1. Figure S1.** The gene expression data distribution of the TARGET (A) and GEO (B) databases. **Figure S2.** Typical images of THP-1 cells, M0 macrophages, and M2 macrophages. **Figure S3.** The efficiency of ST3GAL4 overexpression plasmid in this study. **Figure S4.** The efficiency of ST3GAL4 siRNAs in this study. **Figure S5.** Differences of the overall immune infiltration (A) and 28 immune cells infiltration (B) between high and low metabolism groups. **Figure S6.** The correlation between various immune features in osteosarcoma. **Figure S7.** Differences of the expression of immune checkpoints (A) and core biological pathway activity (B) between high and low metabolism groups. **Figure S8.** PPI network and hub genes in the carbohydrate metabolic pathway. **Figure S9.** PPI network and hub genes in the energy metabolic pathway. **Figure S10.** PPI network and hub genes in the lipid metabolic pathway. **Figure S11.** PPI network and hub genes in the amino acid metabolic pathway. **Figure S12.** PPI network and hub genes in the nucleotide metabolic pathway. **Figure S13.** PPI network and hub genes in the TCA cycle metabolic pathway. **Figure S14.** Metabolic pathway-related clusters based on seven metabolic super-pathways and the relationship between clusters and TIME in osteosarcoma. **Figure S15.** LASSO regression of 114 prognosis-related MRGs. **Figure S16.** Correlations among 17 MRGs of the risk model in the TARGET cohort. **Figure S17.** The heatmap of 28 immune cells between high and low risk groups. **Figure S18.** Correlations between risk score and overall immune infiltration (A), 28 immune cells infiltration (B), and the expression of immune checkpoints (C) and differences of core biological pathway activity between high and low risk score (D). **Figure S19.** Feature plots and violin plots for the core MRGs in the risk model. **Figure S20.** The relationships between ST3GAL4 and CAF score, TIDE score, and dysfunction and exclusion of CTLs. **Figure S21.** The relationships between ST3GAL4 and sensitivities to cisplatin, cyclophosphamide, gemcitabine and sorafenib. **Figure S22.** The prognosis and immunological features of other members in the ST3GAL family. **Figure S23.** Correlations of the expression of ST3GAL4 with metabolic super-pathways (A) and differences of metabolic super-pathways activity between high-ST3GAL4 and low-ST3GAL4 groups (B). **Figure S24.** The GSEA results of ST3GAL4 (A) and other core MRGs (B) based on KEGG gene set.

**Additional file 2. Table S1.** Gene sets for seven metabolic super-pathways.

**Additional file 3. Table S2.**  The primers used in the PCR reaction.

**Additional file 4. Table S3.** The 114 representative prognosis-related MRGs were identified by Cox regression analysis.

**Additional file 5. Table S4.**  Correlations between risk score and IC50 of drugs.

## Data Availability

The datasets analyzed during the current study are available in the TARGET database (https://ocg.cancer.gov/programs/target) and GEO database (https://www.ncbi.nlm.nih.gov/geo/). Further information is available from the corresponding author on reasonable request.

## Abbreviations

TIME: Tumor immune microenvironment
MRG: Metabolism-related gene
TCA: Tricarboxylic acid
GSVA: Gene set variation analysis
GSEA: Gene set enrichment analysis
ssGSEA: Single sample gene set enrichment analysis
OS: Overall survival
RFS: Relapse-free survival
PPI: Protein–protein interaction
GO: Gene ontology
KEGG: Kyoto encyclopedia of genes and genomes
GDSC: Genomics of drug sensitivity in cancer
ICB: Immune checkpoint blockade
CTL: Tumor-infiltrating cytotoxic T lymphocyte
CAF: Tumor-associated fibroblast
MDSC: Myeloid-derived suppressor cell
TAM_M2: M2 tumor-associated macrophage
scRNA-seq: Single-cell RNA-sequencing
IHC: Immunohistochemistry
ST3GAL4: ST3 beta-galactoside alpha-2,3-sialyltransferase 4
TISCH: Tumor immune single-cell hub
APO: Apolipoprotein
FGFR3: Fibroblast growth factor receptor 3
EMT: Epithelial mesenchymal transition
ROC: Receiver operating characteristic
AUC: Area under the time-dependent ROC curve
IC_50_: Half-maximal inhibitory concentration
HIF-1α: Hypoxia inducible factor 1 subunit alpha
LASSO: Least absolute shrinkage and selection operator
HAVCR2: Hepatitis A virus cellular receptor 2
CTLA4: Cytotoxic T-lymphocyte associated protein 4
PDCD1LG2: Programmed cell death 1 ligand 2
IDO1: Indoleamine 2,3-dioxygenase 1
PDCD1: Programmed cell death 1
LAG3: Lymphocyte activating 3
HSPG2: Heparan sulfate proteoglycan 2
AGRN: Agrin
SDC1: Syndecan 1
MTCH2: Mitochondrial carrier 2
ECAR: Extracellular acidification rate
