# Supplementary material for: Exploring the relationship between metabolism and immune microenvironment in osteosarcoma based on metabolic pathways
Source: J Biomed Sci. 2024 Jan 12;31:4. doi: 10.1186/s12929-024-00999-7 (PMC10785352; doi:10.1186/s12929-024-00999-7)
Supplement: Supplementary file 1 — Additional file 1. Figure S1. The gene expression data distribution of the TARGET (A) and GEO (B) databases. Figure S2. Typical images of THP-1 cells, M0 macrophages, and M2 macrophages. Figure S3. The efficiency of ST3GAL4 overexpression plasmid in this study. Figure S4. The efficiency of ST3GAL4 siRNAs in this study. Figure S5. Differences of the overall immune infiltration (A) and 28 immune cells infiltration (B) between high and low metabolism groups. Figure S6. The correlation between various immune features in osteosarcoma. Figure S7. Differences of the expression of immune checkpoints (A) and core biological pathway activity (B) between high and low metabolism groups. Figure S8. PPI network and hub genes in the carbohydrate metabolic pathway. Figure S9. PPI network and hub genes in the energy metabolic pathway. Figure S10. PPI network and hub genes in the lipid metabolic pathway. Figure S11. PPI network and hub genes in the amino acid metabolic pathway. Figure S12. PPI network and hub genes in the nucleotide metabolic pathway. Figure S13. PPI network and hub genes in the TCA cycle metabolic pathway. Figure S14. Metabolic pathway-related clusters based on seven metabolic super-pathways and the relationship between clusters and TIME in osteosarcoma. Figure S15. LASSO regression of 114 prognosis-related MRGs. Figure S16. Correlations among 17 MRGs of the risk model in the TARGET cohort. Figure S17. The heatmap of 28 immune cells between high and low risk groups. Figure S18. Correlations between risk score and overall immune infiltration (A), 28 immune cells infiltration (B), and the expression of immune checkpoints (C) and differences of core biological pathway activity between high and low risk score (D). Figure S19. Feature plots and violin plots for the core MRGs in the risk model. Figure S20. The relationships between ST3GAL4 and CAF score, TIDE score, and dysfunction and exclusion of CTLs. Figure S21. The relationships between ST3GAL4 and sensitivities to c [file 12929_2024_999_MOESM1_ESM.pdf]

Supplementary Figures

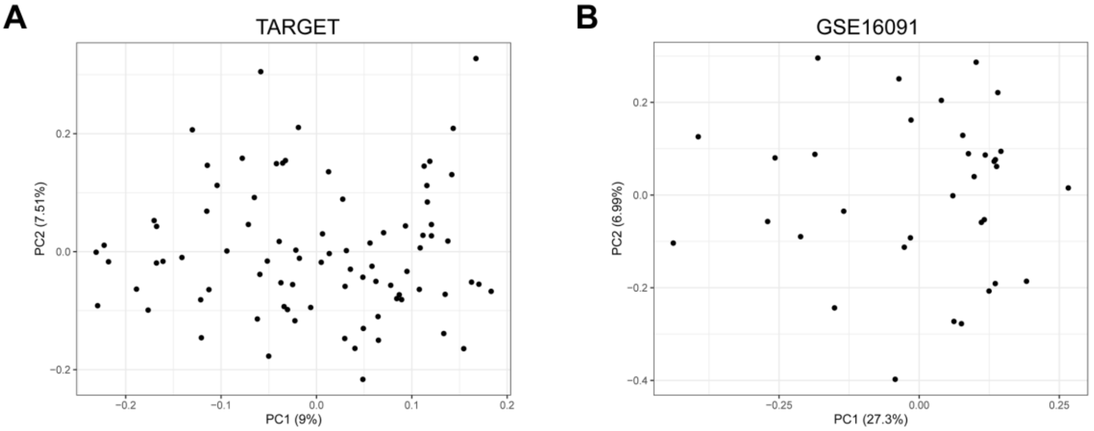

Figure S1. The gene expression data distribution of the TARGET (A) and GEO (B) databases.

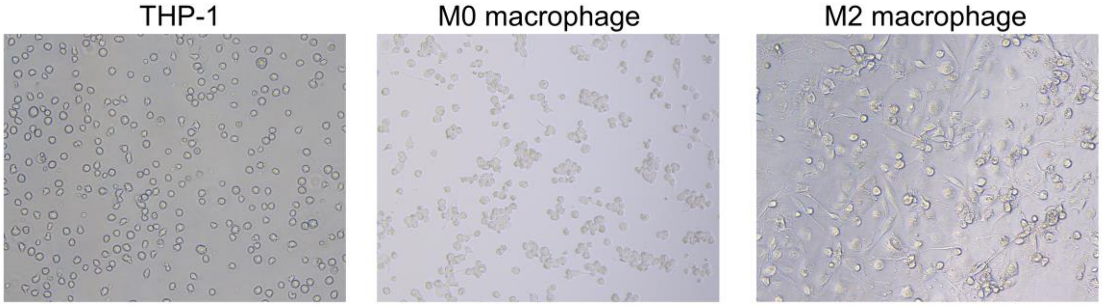

Figure S2. Typical images of THP-1 cells, M0 macrophages, and M2 macrophages.

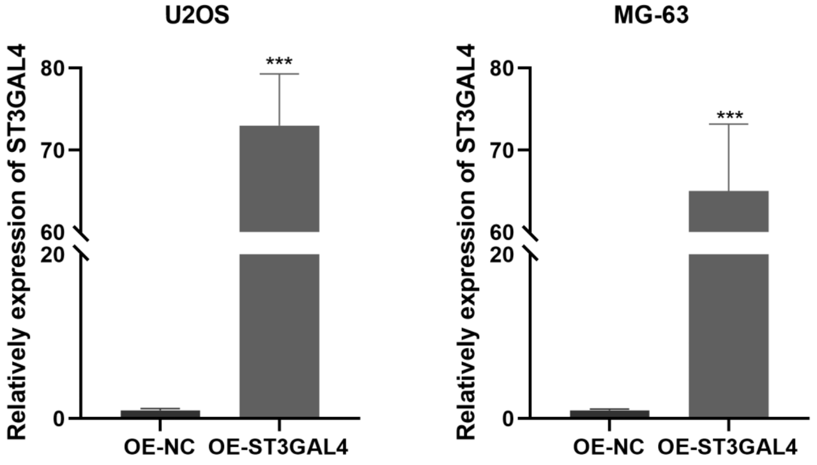

Figure S3. The efficiency of ST3GAL4 overexpression plasmid in this study.

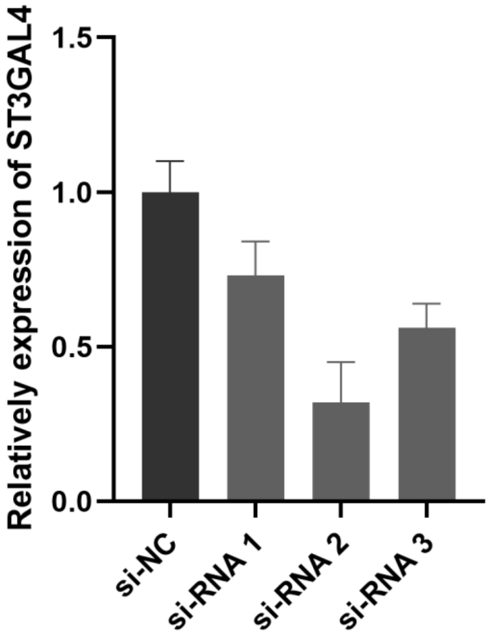

Figure S4. The efficiency of ST3GAL4 siRNAs in this study.

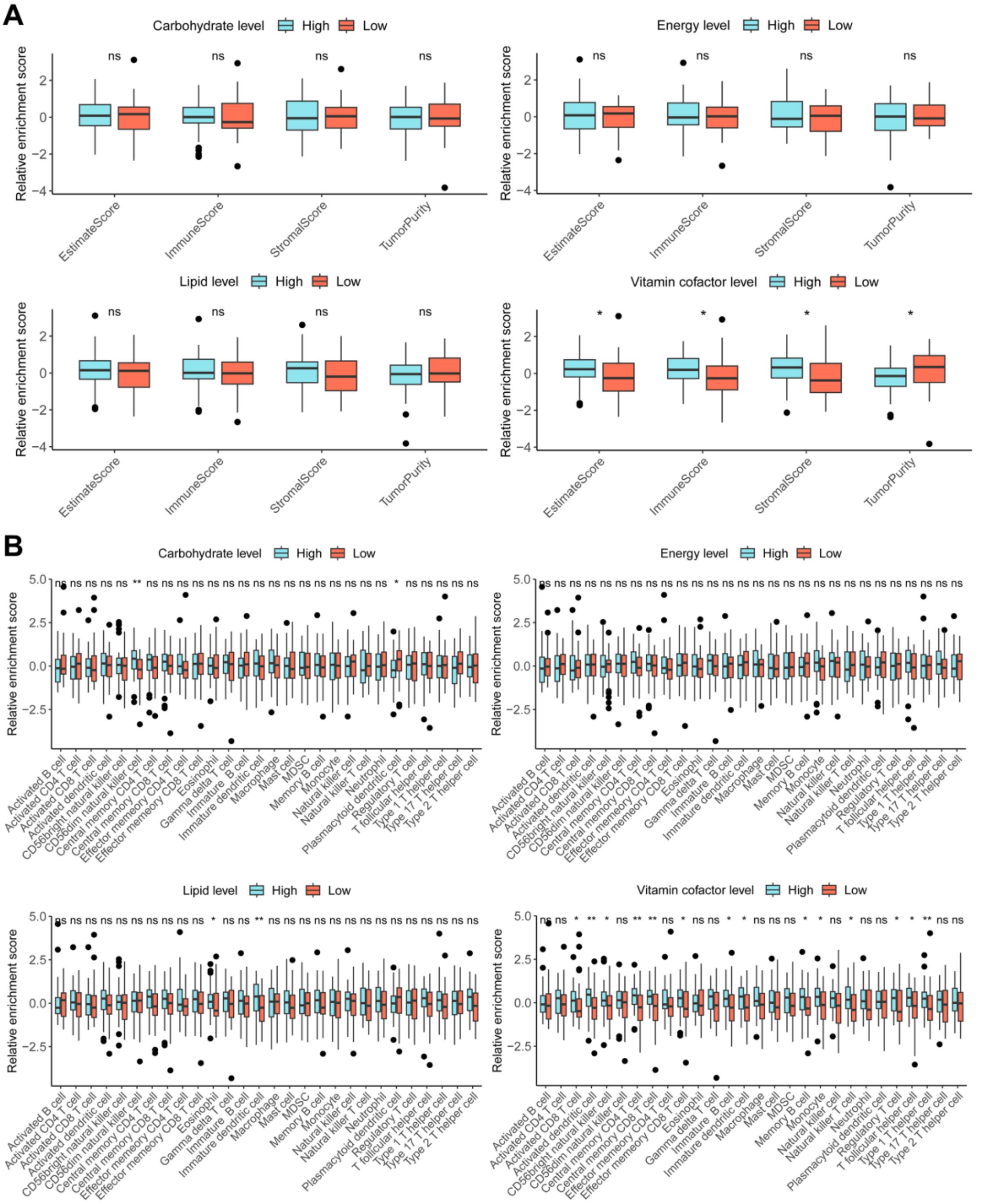

Figure S5. Differences of the overall immune infiltration (A) and 28 immune cells infiltration (B) between high and low metabolism groups.

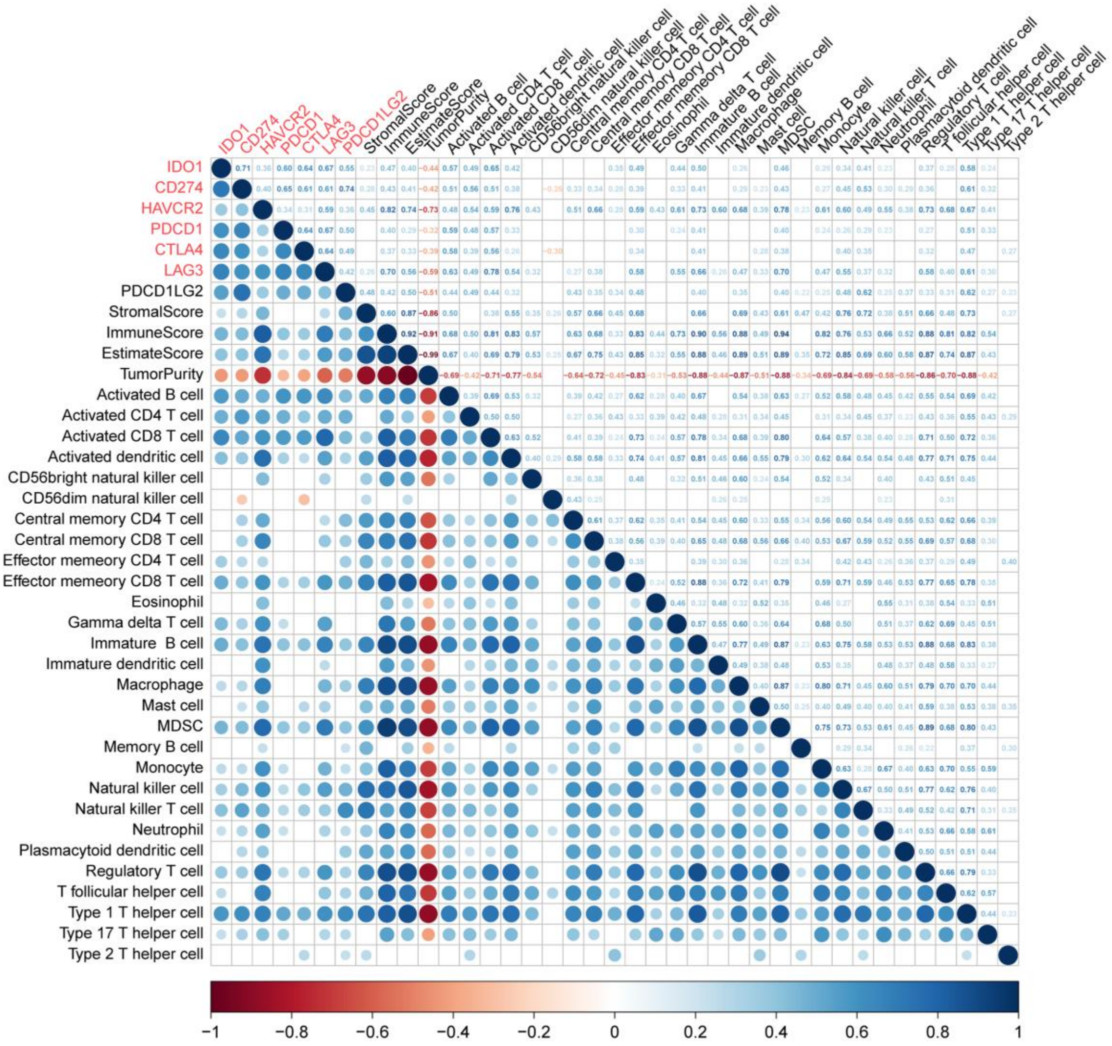

Figure S6. The correlation between various immune features in osteosarcoma.

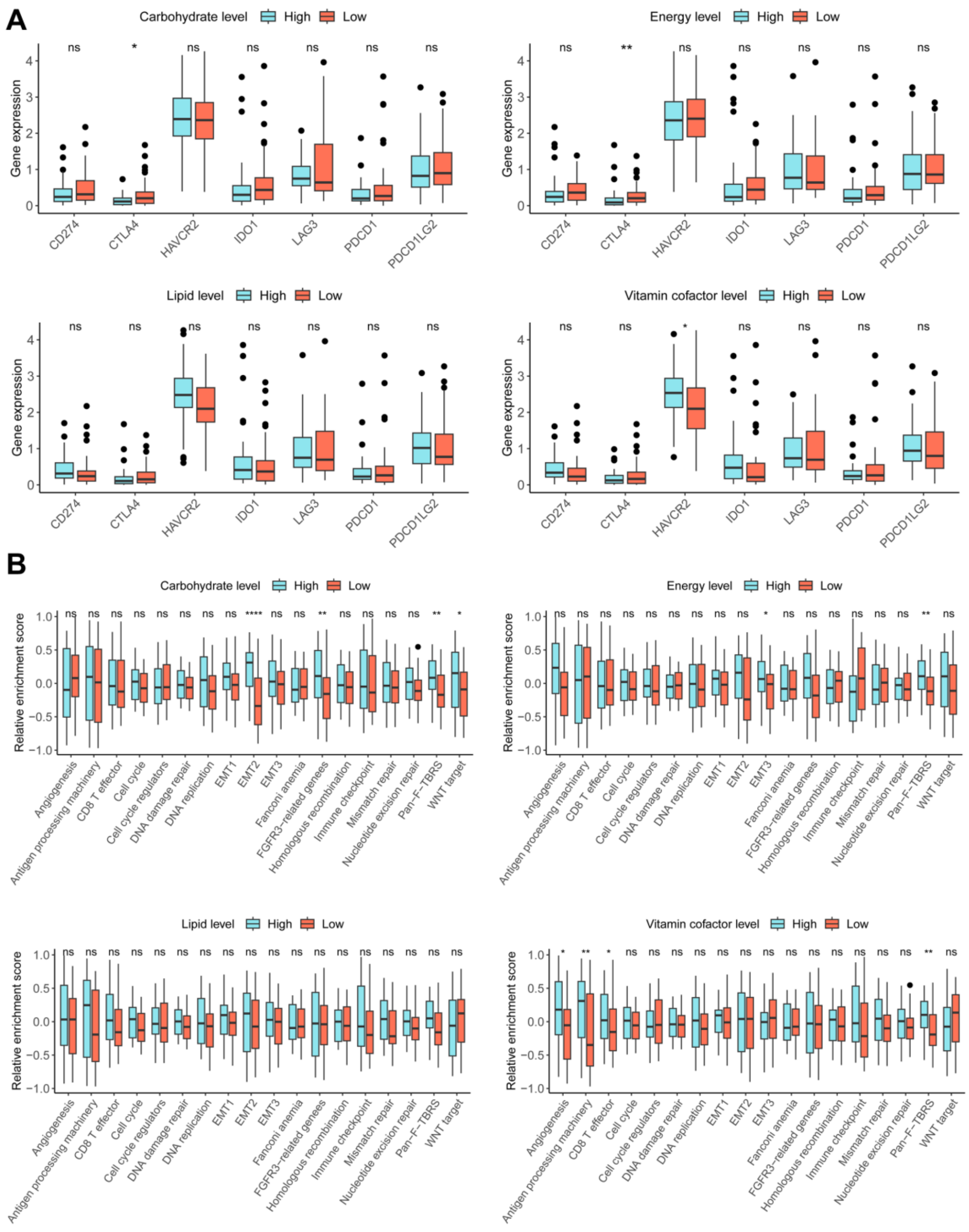

Figure S7. Differences of the expression of immune checkpoints (A) and core biological pathway activity (B) between high and low metabolism groups.

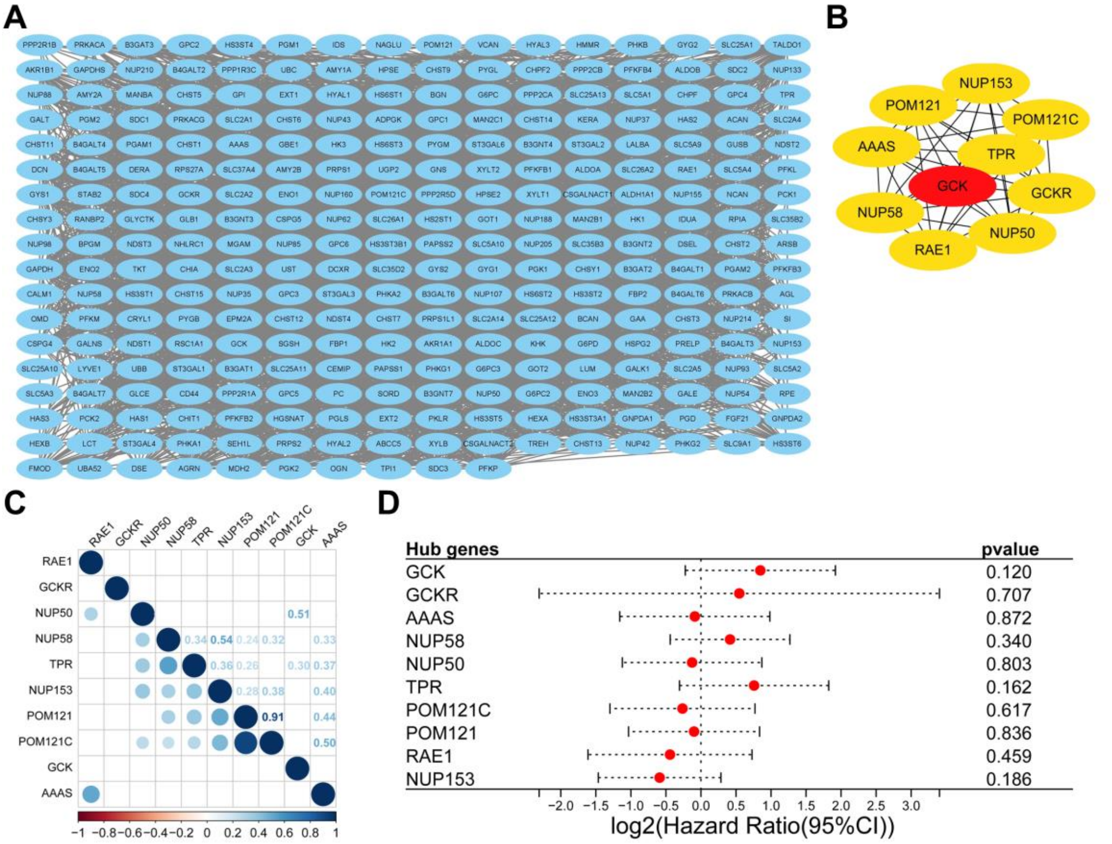

Figure S8. PPI network and hub genes in the carbohydrate metabolic pathway. A PPI network of carbohydrate metabolic pathway genes according to the STRING database. **B** The top 10 hub genes of carbohydrate metabolic pathway genes. **C** Correlations among hub genes in the TARGET cohort. Red representing negative correlations and blue representing positive correlations. Blank represents a correlation P-value > 0.05. **D** Univariate Cox regression analysis of overall survival for hub genes.

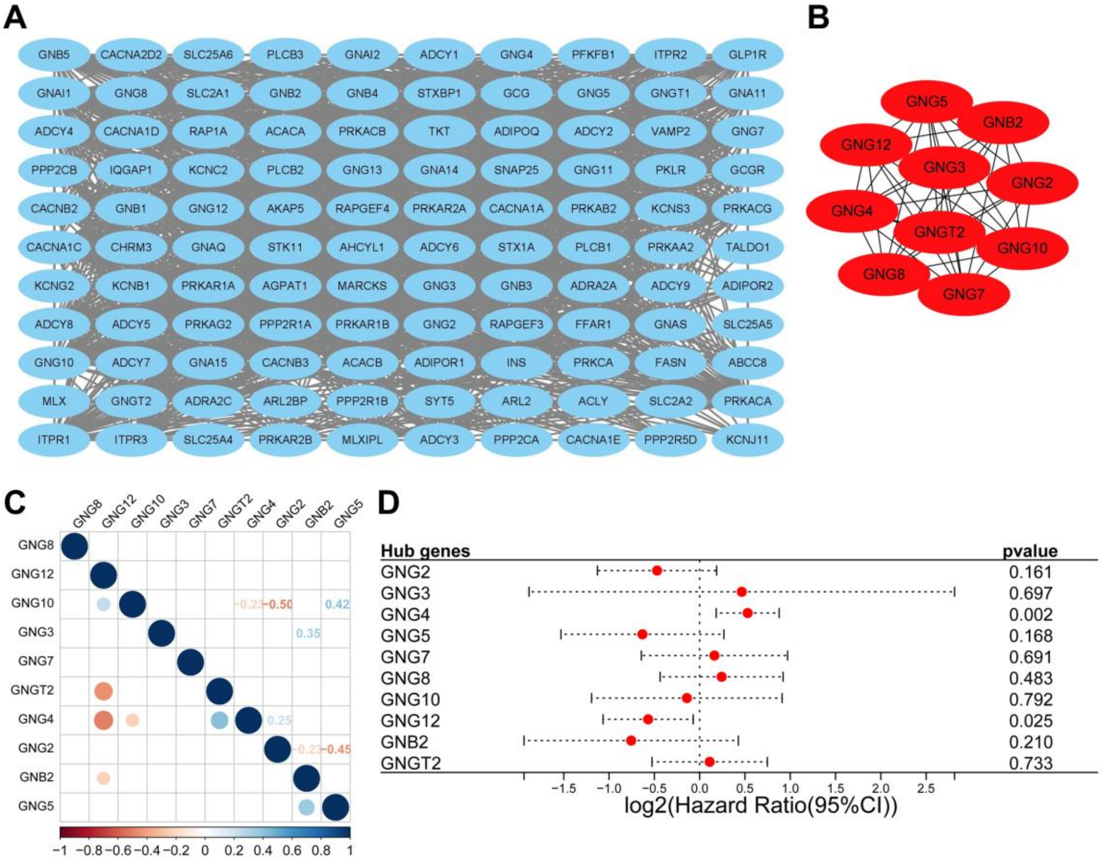

Figure S9. PPI network and hub genes in the energy metabolic pathway. A PPI network of energy metabolic pathway genes according to the STRING database. **B** The top 10 hub genes of energy metabolic pathway genes. **C** Correlations among hub genes in the TARGET cohort. Red representing negative correlations and blue representing positive correlations. Blank represents a correlation P-value > 0.05. **D** Univariate Cox regression analysis of overall survival for hub genes.

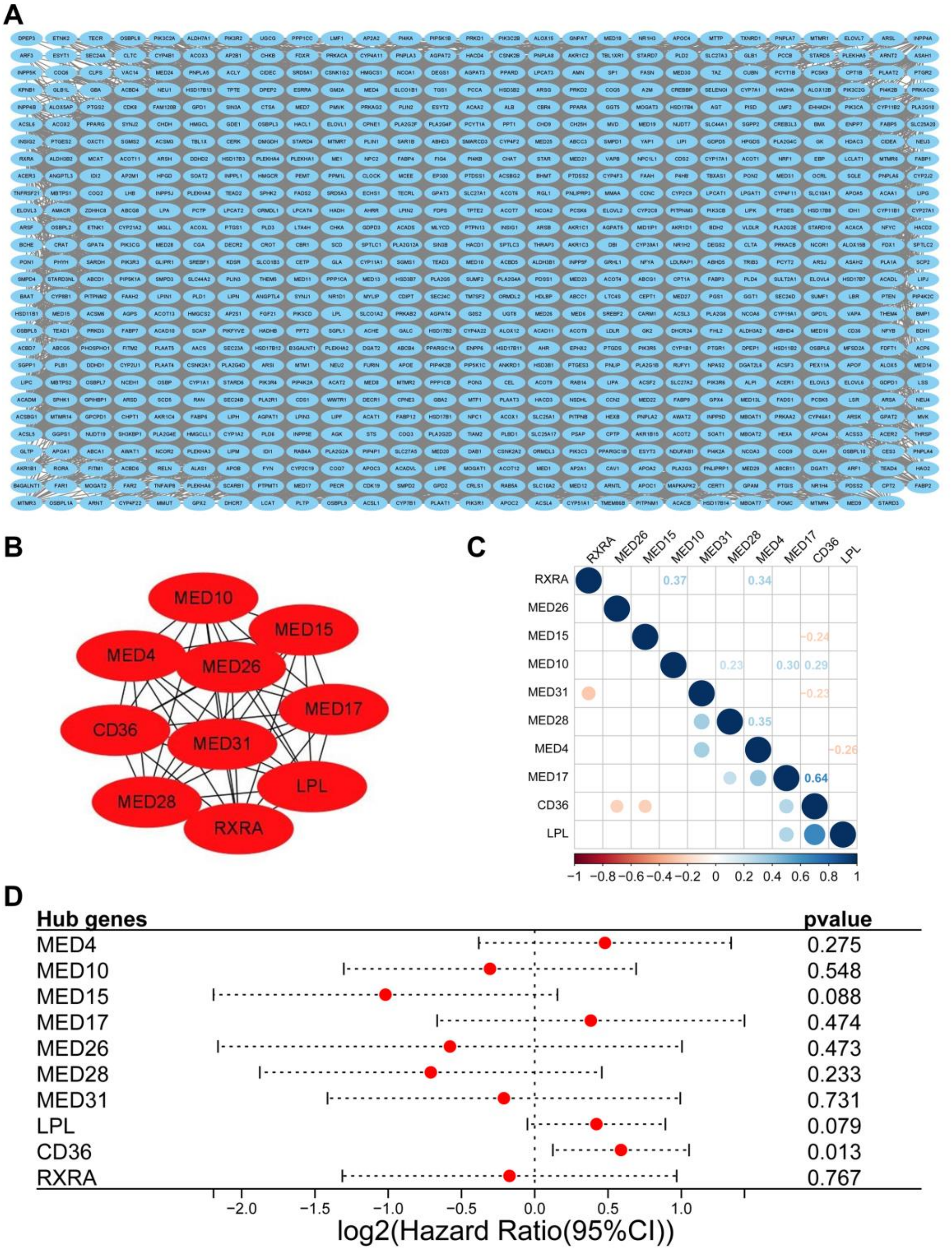

Figure S10. PPI network and hub genes in the lipid metabolic pathway. A PPI network of lipid metabolic pathway genes according to the STRING database. **B** The top 10 hub genes of lipid metabolic pathway genes. **C** Correlations among hub genes in the TARGET cohort. Red representing negative correlations and blue representing positive correlations. Blank represents a correlation P-value > 0.05. **D** Univariate Cox regression analysis of overall survival for hub genes.

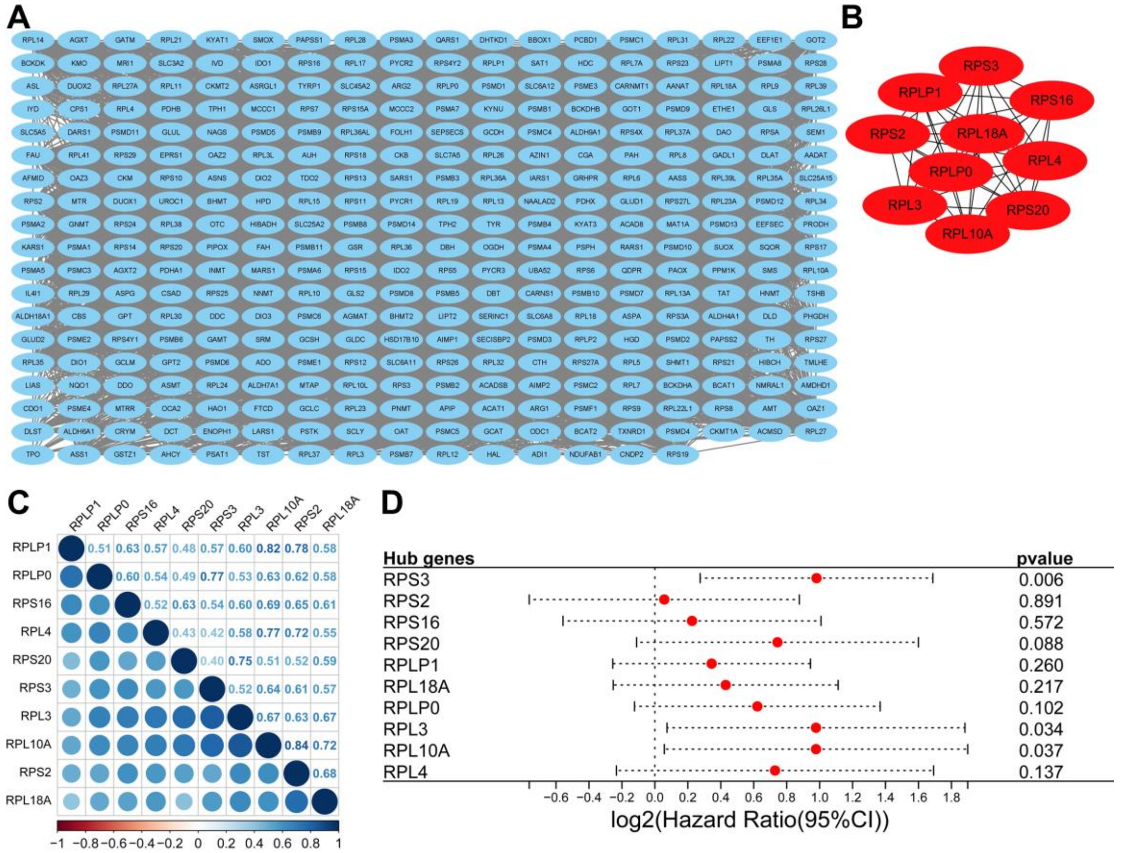

Figure S11. PPI network and hub genes in the amino acid metabolic pathway. A PPI network of amino acid metabolic pathway genes according to the STRING database. **B** The top 10 hub genes of amino acid metabolic pathway genes. **C** Correlations among hub genes in the TARGET cohort. Red representing negative correlations and blue representing positive correlations. Blank represents a correlation P-value > 0.05. **D** Univariate Cox regression analysis of overall survival for hub genes.

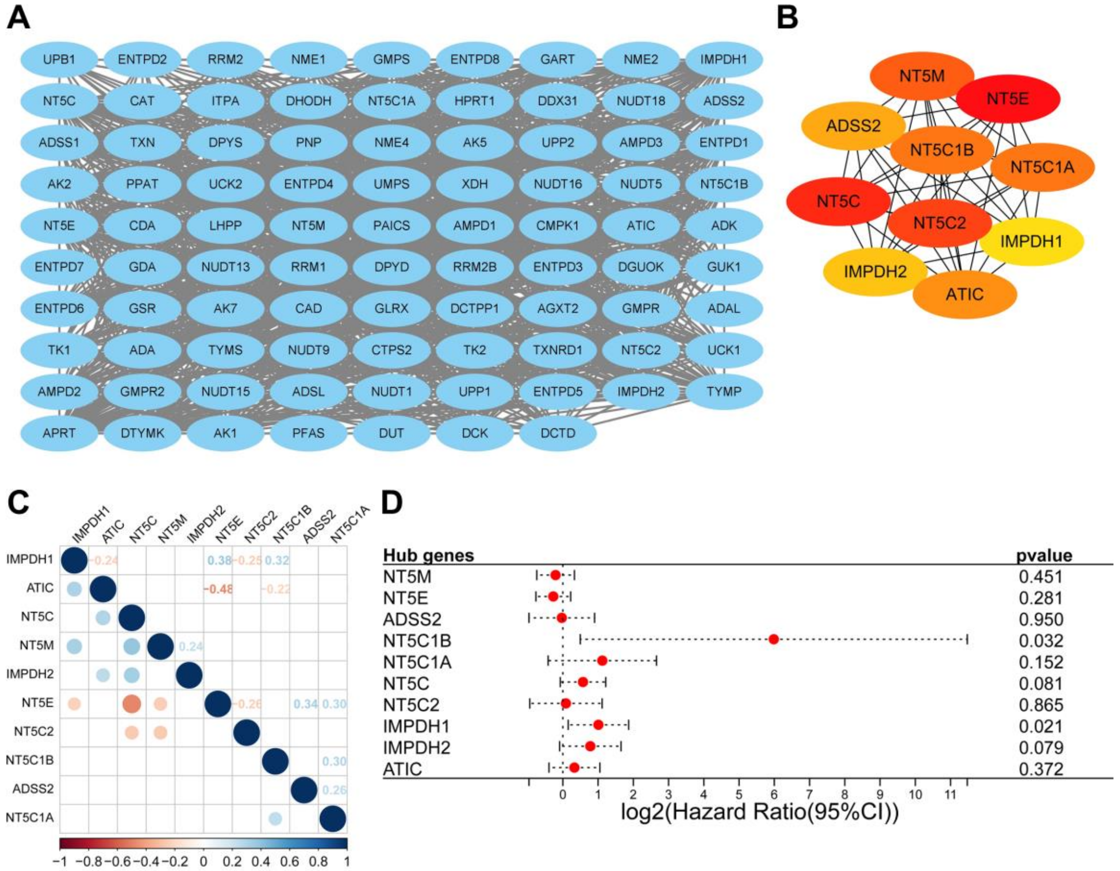

Figure S12. PPI network and hub genes in the nucleotide metabolic pathway. A PPI network of nucleotide metabolic pathway genes according to the STRING database. **B** The top 10 hub genes of nucleotide metabolic pathway genes. **C** Correlations among hub genes in the TARGET cohort. Red representing negative correlations and blue representing positive correlations. Blank represents a correlation P-value > 0.05. **D** Univariate Cox regression analysis of overall survival for hub genes.

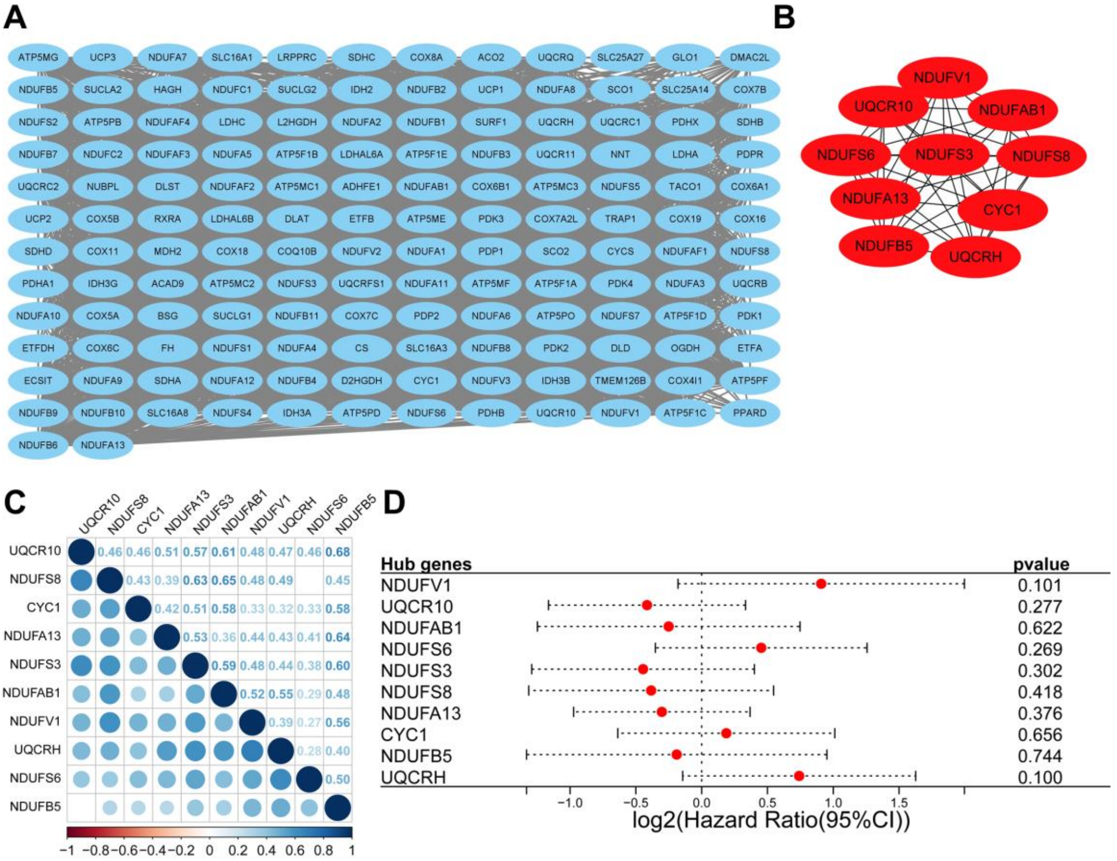

Figure S13. PPI network and hub genes in the TCA cycle metabolic pathway. A PPI network of TCA cycle metabolic pathway genes according to the STRING database. **B** The top 10 hub genes of TCA cycle metabolic pathway genes. **C** Correlations among hub genes in the TARGET cohort. Red representing negative correlations and blue representing positive correlations. Blank represents a correlation P-value > 0.05. **D** Univariate Cox regression analysis of overall survival for hub genes.

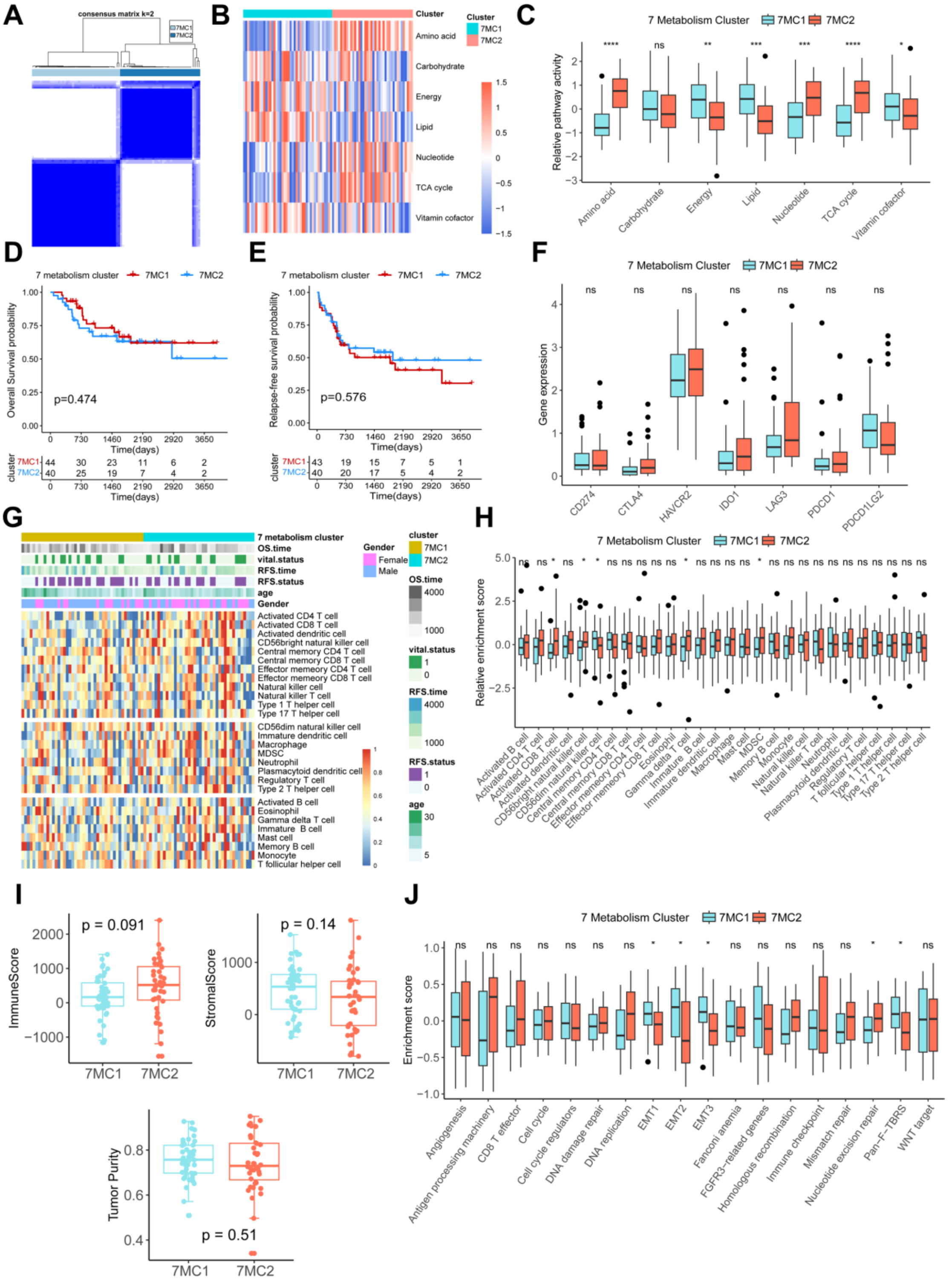

Figure S14. Metabolic pathway-related clusters based on seven metabolic super-pathways and the relationship between clusters and TIME in osteosarcoma. A Consensus heatmap based on four key metabolic pathways in the TARGET cohort. **B** The heatmap of four key metabolic pathways between 7MC1 and 7M C2. **C** Differences

of four key metabolic pathways between 7MC1 and 7MC2. **D**,**E** Kaplan-Meier curves depict the OS (**D**) and RFS (**E**) difference between 7MC1 and 7MC2. Red representing the 7MC1 patients and blue representing the 7MC2 patients. **F** Differences of immune checkpoint genes expression between 7MC1 and 7MC2. **G** The heatmap of 28 immune cells between the two clusters and the correlations of the clusters and clinical parameters. **H** Differences of the abundance of 28 immune cells between 7MC1 and 7MC2. **I** Differences of ImmuneScore, StromalScore and tumor purity between 7MC1 and 7MC2. **J** Differences of core biological pathway activity between 7MC1 and 7MC2. * $P < 0.05$, ** $P < 0.01$, **** $P < 0.0001$.

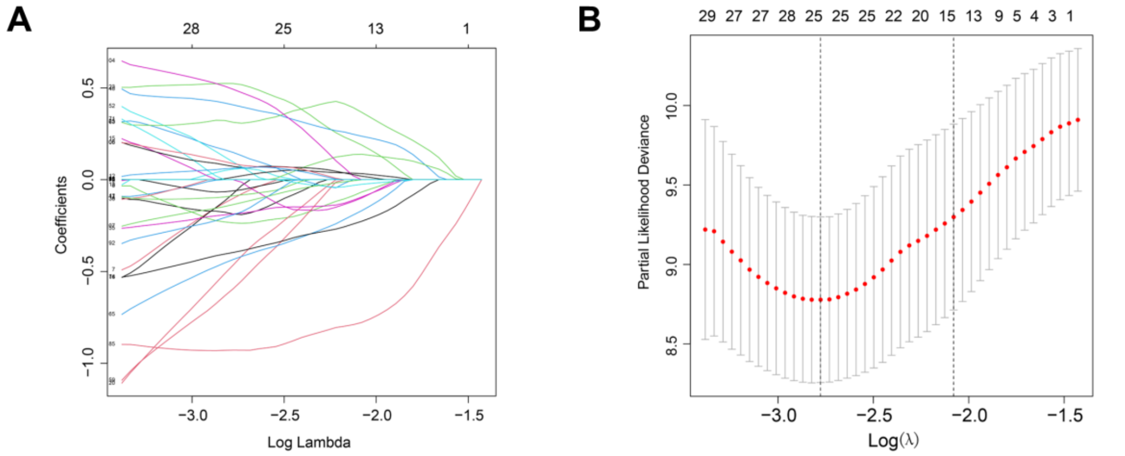

Figure S15. LASSO regression of 114 prognosis-related MRGs. A LASSO coefficient profiles of 25 prognostic MRGs. **B** Ten-time cross-validation for tuning parameter selection in the LASSO model.

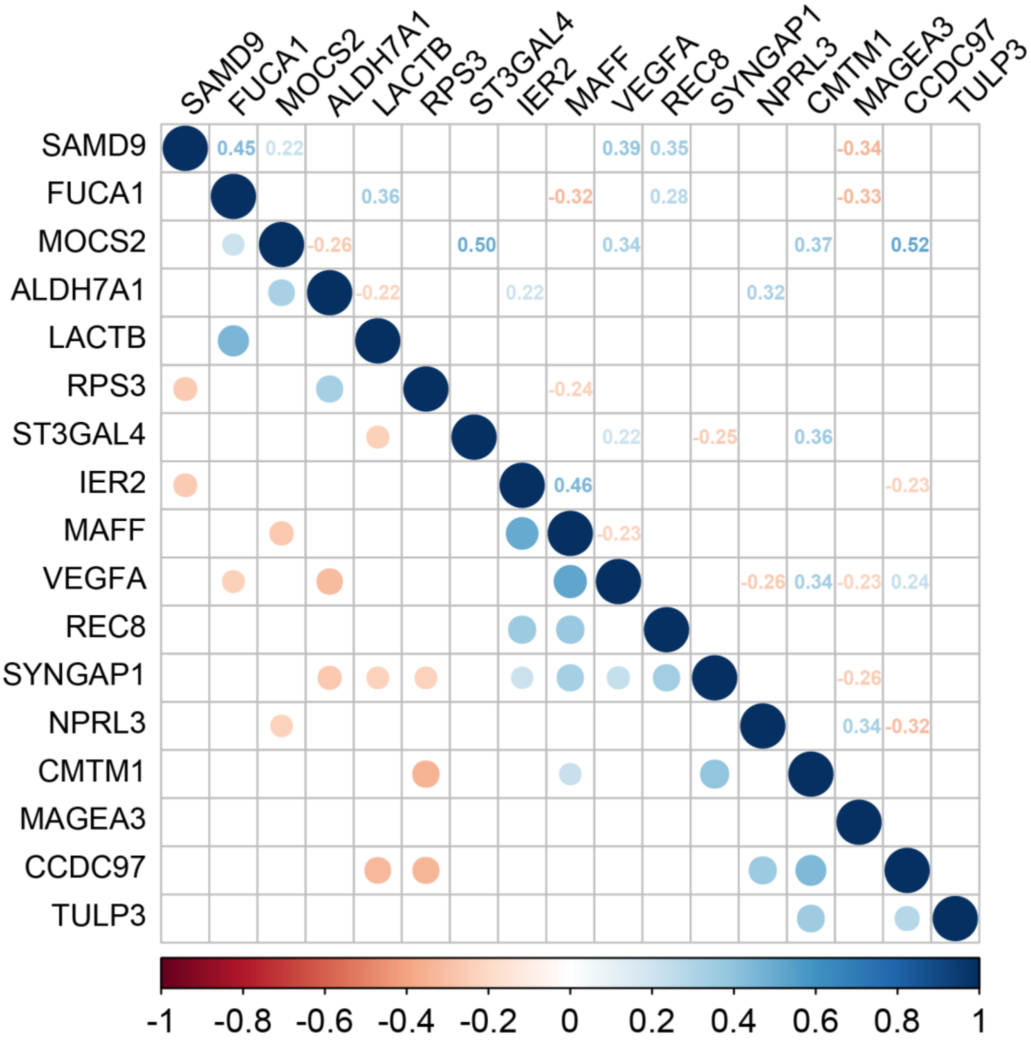

Figure S16. Correlations among 17 MRGs of the risk model in the TARGET cohort. Red representing negative correlations and blue representing positive correlations. Blank represents a correlation P-value > 0.05.

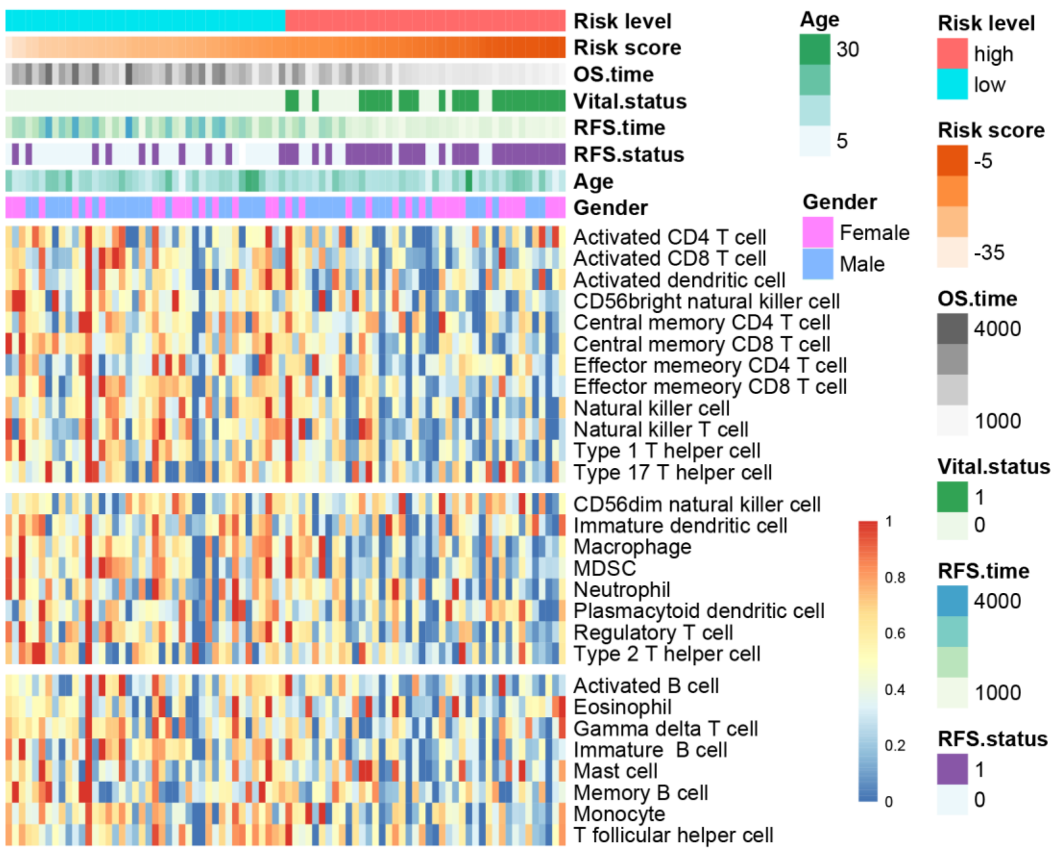

Figure S17. The heatmap of 28 immune cells between high and low risk groups.

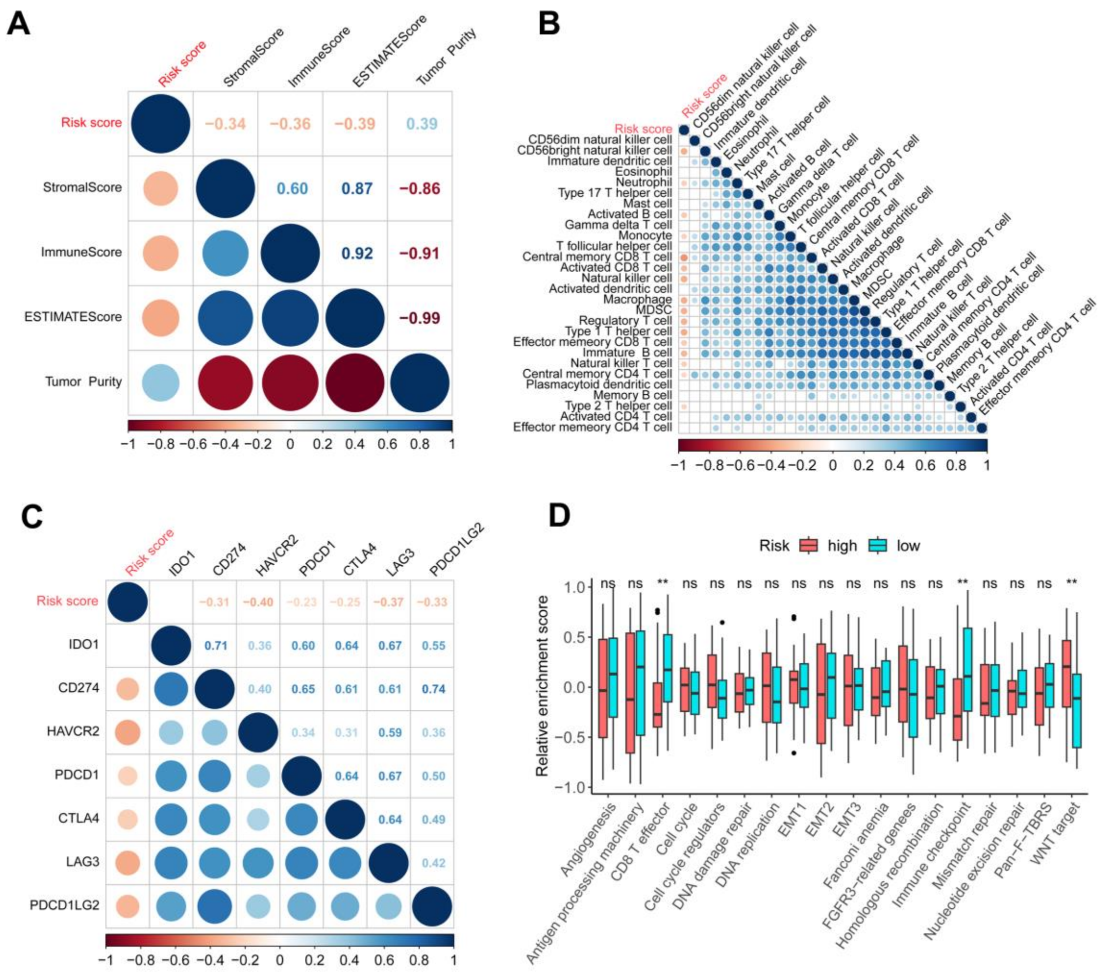

Figure S18. Correlations between risk score and overall immune infiltration (A), 28 immune cells infiltration (B), and the expression of immune checkpoints (C) and differences of core biological pathway activity between high and low risk score (D).

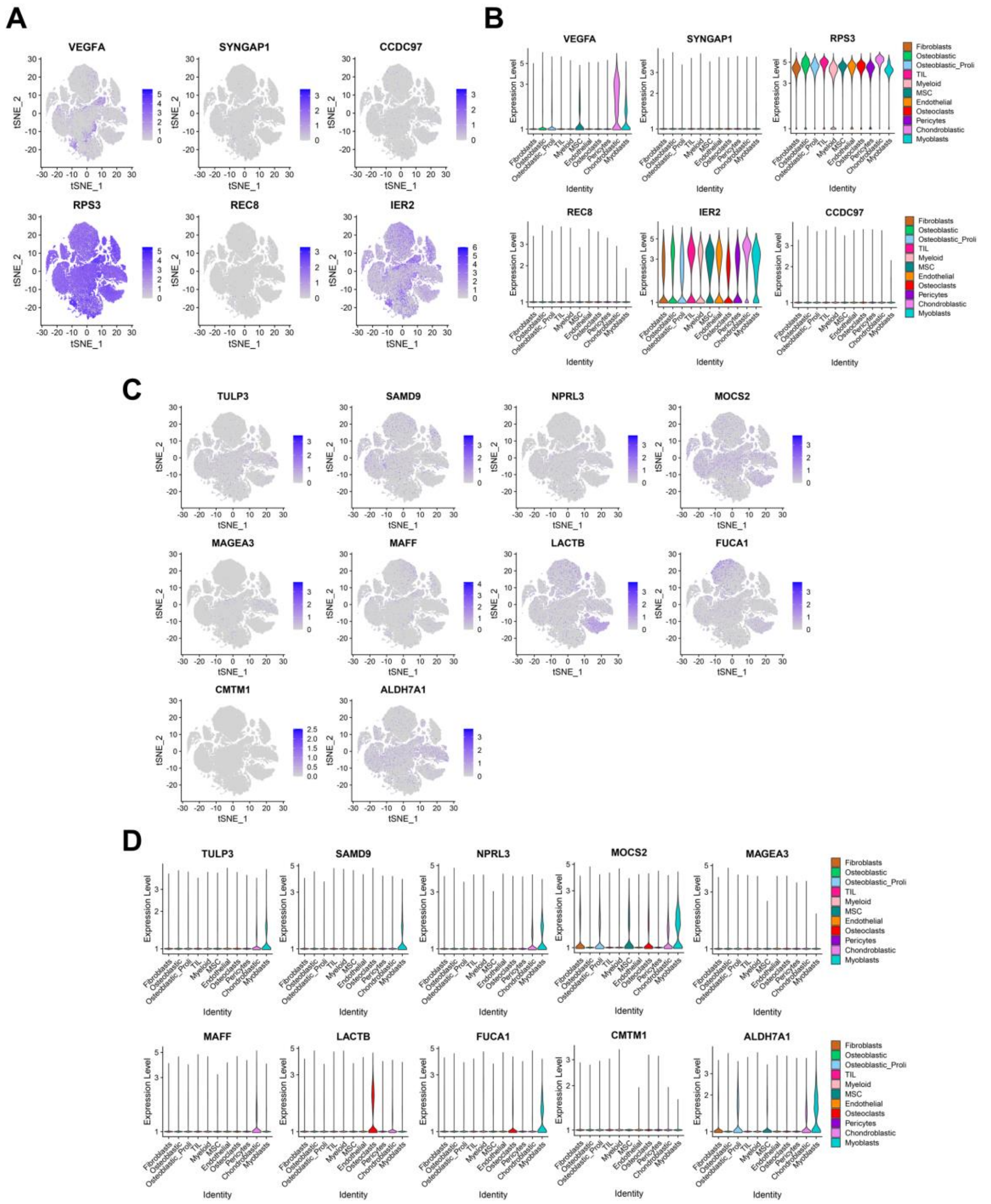

Figure S19. Feature plots and violin plots for the core MRGs in the risk model.

A,B Feature plots and violin plots of MRGs with positive coefficients in the risk model.

C,D Feature plots and violin plots of MRGs with negative coefficients in the risk model.

The color legend shows the normalized expression levels of the genes.

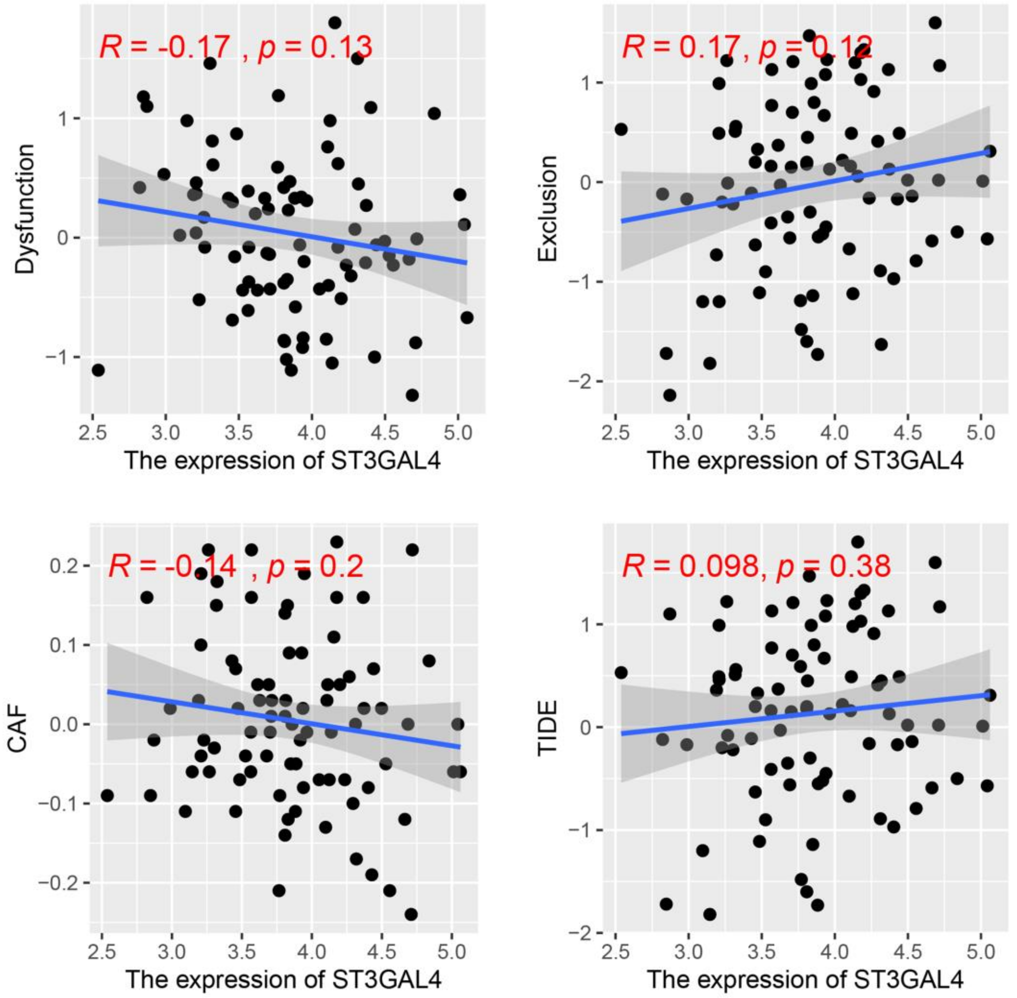

Figure S20. The relationships between ST3GAL4 and CAF score, TIDE score, and dysfunction and exclusion of CTLs.

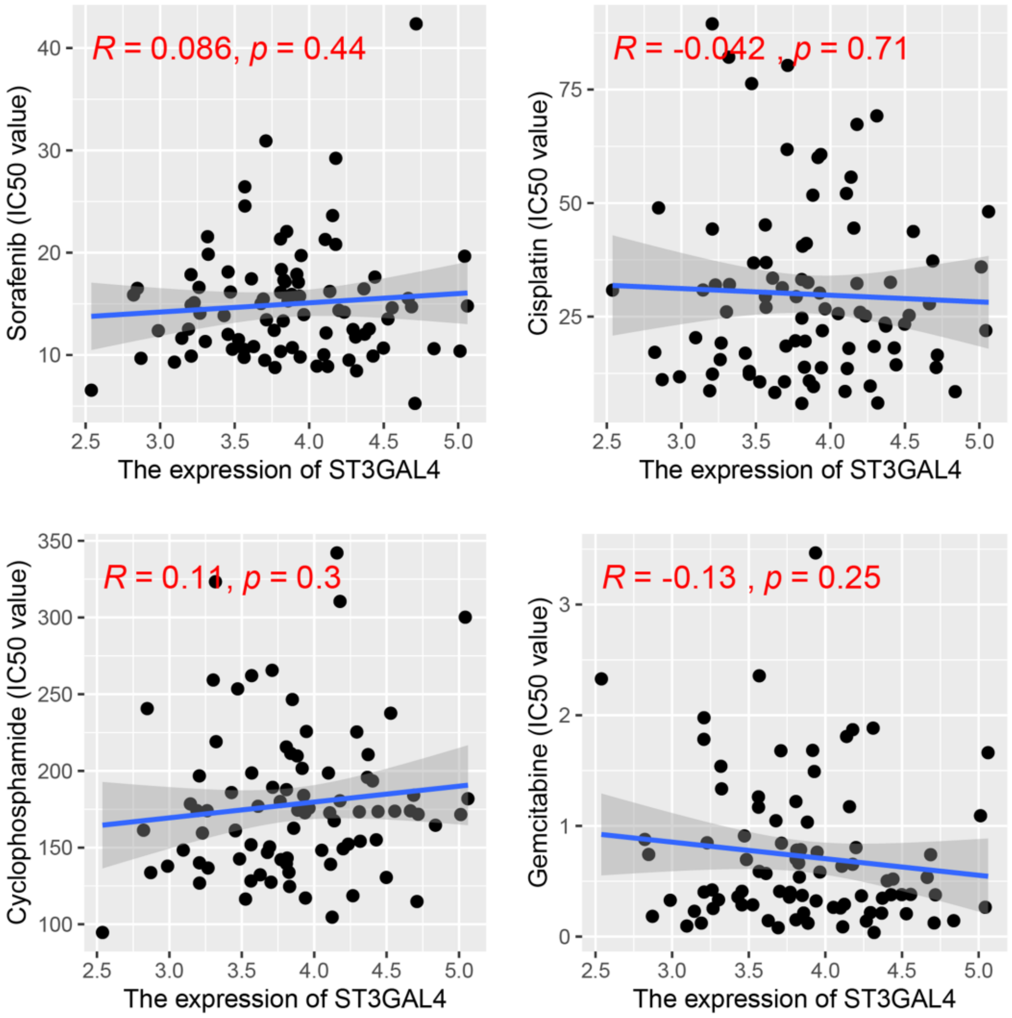

Figure S21. The relationships between ST3GAL4 and sensitivities to cisplatin, cyclophosphamide, gemcitabine and sorafenib.

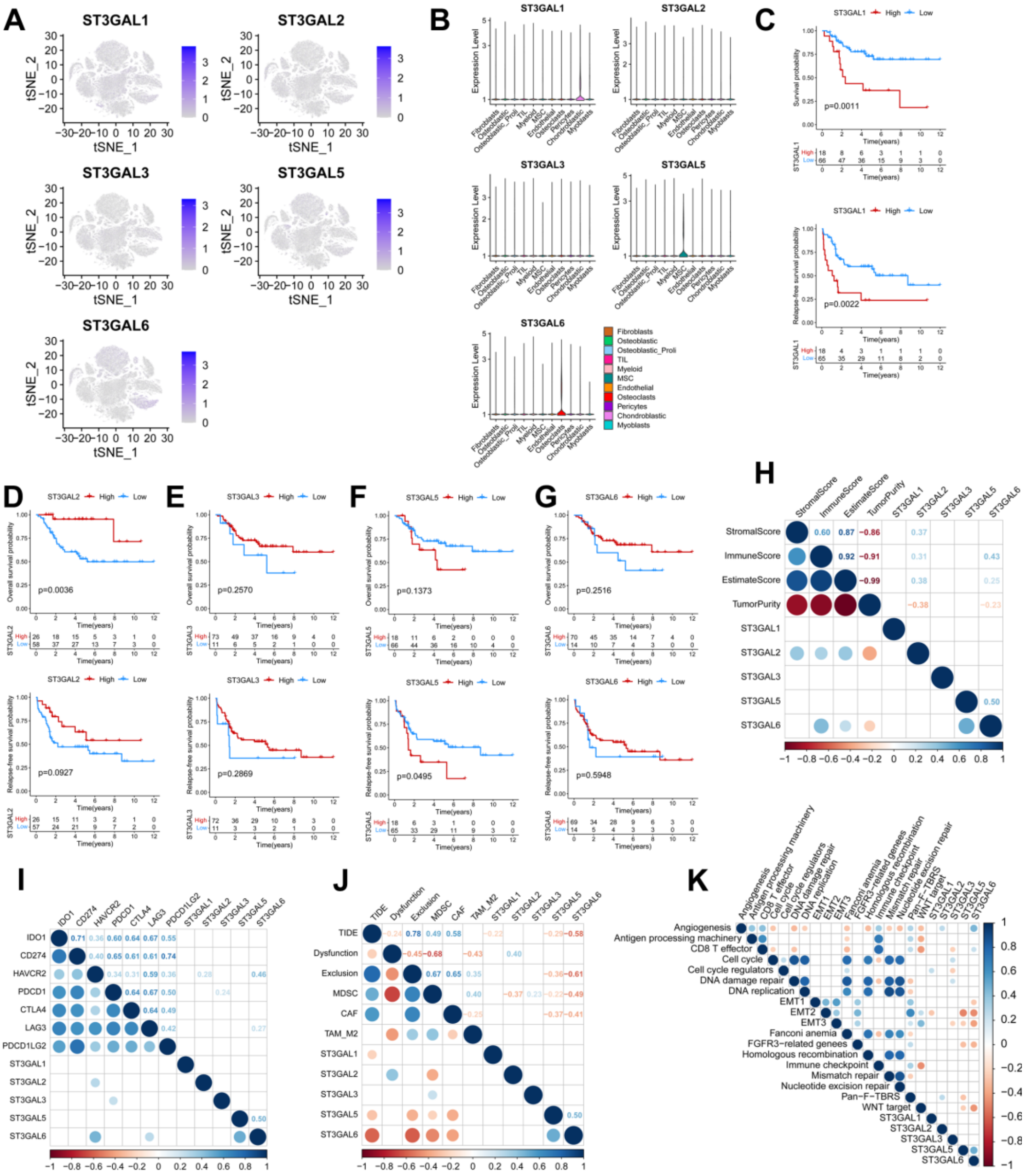

Figure S22. The prognosis and immunological features of other members in the ST3GAL family. A,B Feature plots **(A)** and violin plots **(B)** for the members of ST3GAL family. **C-G** Kaplan-Meier curves depict the OS and RFS difference between high and low ST3GAL groups. **H-K** Correlations of the expression of ST3GAL family with overall immune infiltration **(H)**, immune checkpoints **(I)**, TIDE scores **(J)**, and known core biological pathway scores **(K)**. Correlation coefficients are calculated by

Spearman’s correlation analysis, with red representing negative correlations and blue representing positive correlations. Blank represents a correlation P-value > 0.05.

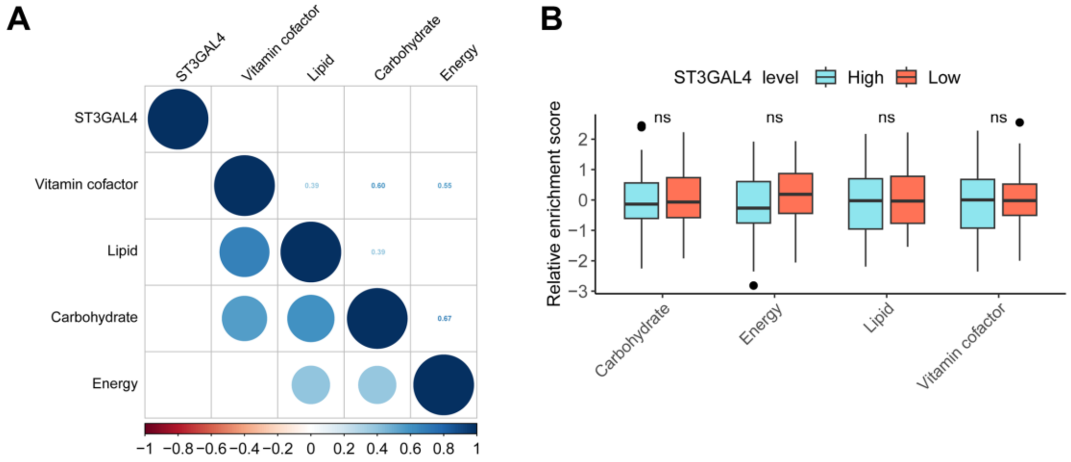

Figure S23. Correlations of the expression of ST3GAL4 with metabolic super-pathways (A) and differences of metabolic super-pathways activity between high-ST3GAL4 and low-ST3GAL4 groups (B).

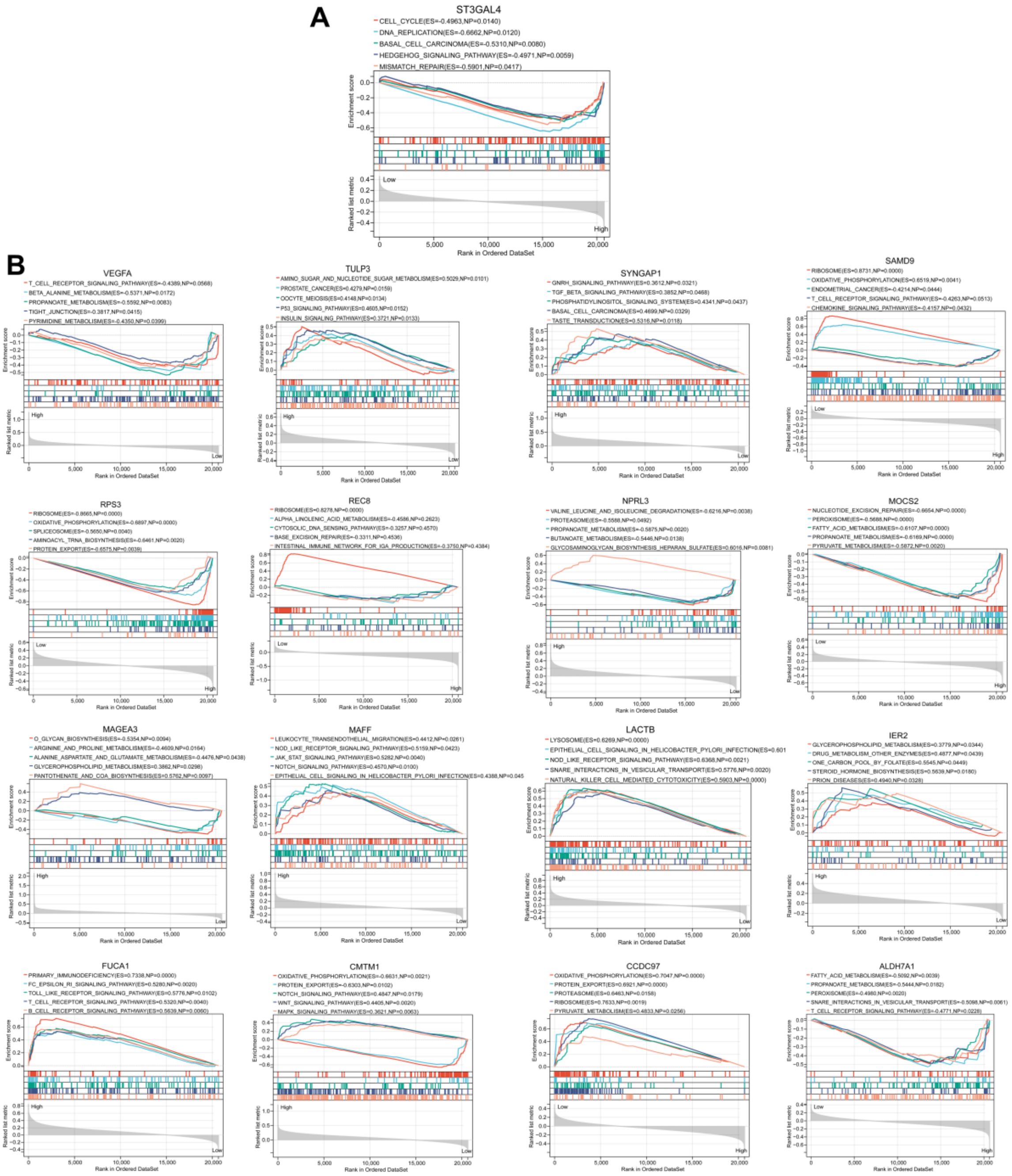

Figure S24. The GSEA results of ST3GAL4 (A) and other core MRGs (B) based on KEGG gene set.
