# Supplementary material for: Exploring the relationship between metabolism and immune microenvironment in osteosarcoma based on metabolic pathways
Source: J Biomed Sci. 2024 Jan 12;31:4. doi: 10.1186/s12929-024-00999-7 (PMC10785352; doi:10.1186/s12929-024-00999-7)
Supplement: Supplementary file 3 — Additional file 3. Table S2. The primers used in the PCR reaction. [file 12929_2024_999_MOESM3_ESM.pdf]

Table S2. The primers used in the PCR reaction

Gene Name	Forward	Reverse
ST3GAL4	CCCATCTTCCTGCGGCTTG	CCTGAGGCTCTGGATGTTCTTG
PD-L1	GCTGCACTAATTGTCTATTGGG	CACAGTAATTCGCTTGTAGTCG
CD206	CACCATCGAGGAATTGGACT	ACAATTCGTCATTTGGCTCA
ACTB	CTACCTCATGAAGATCCTCACCGA	TTCTCCTTAATGTCACGCACGATT
